# Synthesis and Evaluation of Antioxidant Properties of 2-Substituted Quinazolin-4(*3H*)-ones

**DOI:** 10.3390/molecules26216585

**Published:** 2021-10-30

**Authors:** Janez Mravljak, Lara Slavec, Martina Hrast, Matej Sova

**Affiliations:** Faculty of Pharmacy, University of Ljubljana, Aškerčeva cesta 7, SI-1000 Ljubljana, Slovenia; janez.mravljak@ffa.uni-lj.si (J.M.); lara.slavec@gmail.com (L.S.); martina.hrast@ffa.uni-lj.si (M.H.)

**Keywords:** quinazolinone, synthesis, antioxidant, DPPH, ABTS, CUPRAC, metal-chelating properties

## Abstract

Quinazolinones represent an important scaffold in medicinal chemistry with diverse biological activities. Here, two series of 2-substituted quinazolin-4(3*H*)-ones were synthesized and evaluated for their antioxidant properties using three different methods, namely DPPH, ABTS and TEAC_CUPRAC_, to obtain key information about the structure–antioxidant activity relationships of a diverse set of substituents at position 2 of the main quinazolinone scaffold. Regarding the antioxidant activity, ABTS and TEAC_CUPRAC_ assays were more sensitive and gave more reliable results than the DPPH assay. To obtain antioxidant activity of 2-phenylquinazolin-4(3*H*)-one, the presence of at least one hydroxyl group in addition to the methoxy substituent or the second hydroxyl on the phenyl ring in the ortho or para positions is required. An additional ethylene linker between quinazolinone ring and phenolic substituent, present in the second series (compounds **25a** and **25b**), leads to increased antioxidant activity. Furthermore, in addition to antioxidant activity, the derivatives with two hydroxyl groups in the ortho position on the phenyl ring exhibited metal-chelating properties. Our study represents a successful use of three different antioxidant activity evaluation methods to define 2-(2,3-dihydroxyphenyl)quinazolin-4(3*H*)-one **21e** as a potent antioxidant with promising metal-chelating properties.

## 1. Introduction

**Figure 1:**
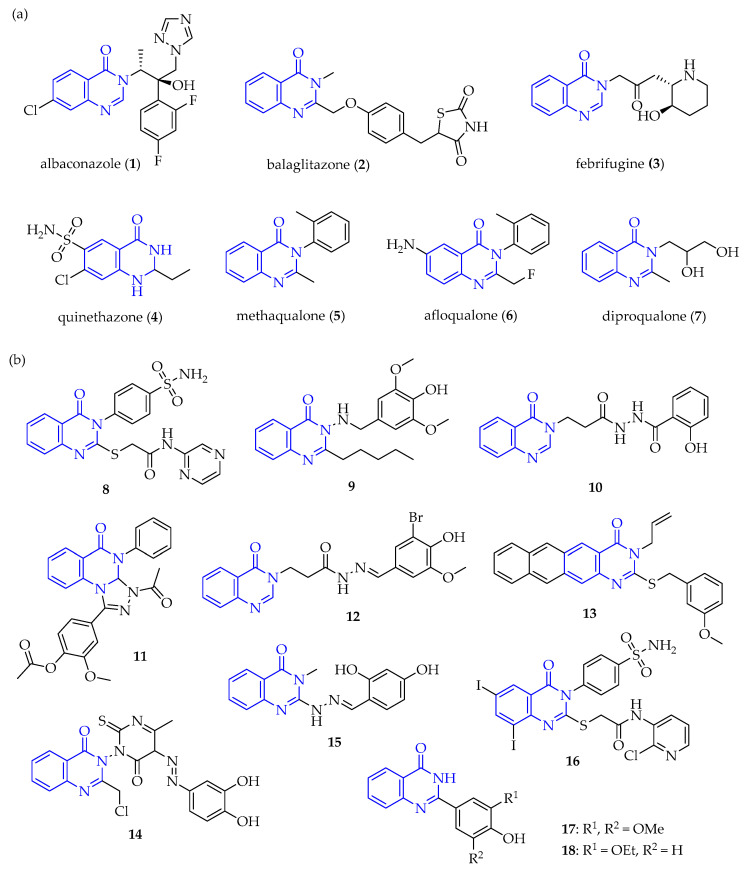
(**a**) Representative quinazolin-4(3*H*)-one-based drugs **1**–**10**; (**b**) representative examples of diverse quinazolin-4(3*H*)-ones **8**–**18** with antioxidant activity.

Quinazoline is a nitrogen containing fused heterocycle, and with additional carbonyl linkage it forms two different quinazolinones (either 4(3*H*)-quinazolinone or 2(1*H*)-quinazolinone) [1]. Quinazolinones represent an important scaffold in medicinal chemistry due to their synthetic accessibility and diverse in vitro and in vivo pharmacological activities [2,3]. Many synthetic and natural quinazoline-based drugs have been used clinically to treat diverse pathological conditions. The most known drugs among 4(3*H*)-quinazolinones are the triazole antifungal drug albaconazole (**1**), the antihyperglicemic agent balaglitazone (**2**), the antimalarial agent febrifugine (**3**), the antihypertensive agent quinethazone (**4**) and the quinazolines with GABAergic activity (e.g., methaqualone (**5**), afloqualone (**6**) and diproqualone (**7**)) (Figure 1a) [1].

The antioxidant properties were described for structurally diverse quinazolines (Figure 1b), such as 2-pentylquinazolin-4(3*H*)-one derivatives (e.g., **8**) [4], heterocyclic quinazoline-4-one derivatives [5,6] (e.g., **9** [6]), *N*-(pyrazin-2-yl)-2-[(4-oxo-3-(4-sulfamoylphenyl)-3,4-dihydroquinazolin-2-yl)thio]acetamide (**10**) [7], quinazolinone-1,3,4-oxadiazole conjugates (e.g., **11**) [8], and a series of 2,3-disubstituted-2,3-dihydro-quinazolin-4(1*H*)-one-derived [9,10] and quinazolinone-derived Schiff′s bases (e.g., **12**) [11,12]. Furthermore, antioxidant activity was also reported for 2-thioxobenzo[*g*]quinazoline derivatives (e.g., **13**) [13], bis(2,3-dihydroquinazolin-4(1*H*)-one derivatives [14], 2-(chloromethyl)-3-(4-methyl-6-oxo-5-[(*E*)-phenyldiazenyl]-2-thioxo-5,6-dihydropyrimidine-1(2*H*)-yl)quinazoline-4(3*H*)-ones (e.g., **14**) [15], guanine-based (*E)*-2-(2-(pyridin-2-ylmethylene)hydrazinyl)quinazolin-4(3*H*)-ones [16], quinazolinone-based hydrazones (e.g., **15**) [17] and iodinated quinazolinones bearing a benzensulfonamide moiety (e.g., **16**) [18].

There are many different analytical methods for assessing the antioxidant capacity. They can be divided into three distinctive groups, namely spectrometry, electrochemical techniques and chromatography (GC and HPLC) [19,20]. Spectrometric techniques are the most commonly used assays because they are rapid, simple and easily accessible. They are based on measuring the change in absorbance or fluorescence of an indicator containing solution after the addition of the antioxidant [21]. As indicators, compounds capable of detecting hydrogen atom transfer (e.g., α,α-azabisizobutronytril, fluorescein, luminol) or single electron transfer (e.g., Cu^2+^, Fe^3+^, 2,2′-azinobis(3-ethylbenzthiazolin-6-sulfonic acid (ABTS), 2,2-di(4-tert-octylphenyl)1-picrylhydrazyl) [20]) are used. A widely used assay for the determination of antioxidant potential is the DPPH (2,2-diphenyl-1-picryl-hydrazyl-hydrate) free radical scavenging method, measuring the reduction of odd electrons on nitrogen atoms in DPPH (by receiving a hydrogen atom from antioxidants) to the corresponding hydrazine [22]. When a transition metal (such as copper) is used as an indicator, the total antioxidant capacity is determined. For example, the commonly used cupric reducing antioxidant capacity (CUPRAC) assay is based on the reduction of cupric (Cu^2+^) to cuprous (Cu^+^) and the absorbance of the formed Cu(I)-neocuproine chelate is measured [23]. On the other hand, cyclic voltammetry and bi/amperometry are the most commonly used electrochemical methods, whereas GC and HPLC are most often applied for antioxidant separation and detection in complex samples [19].

Recently, we discovered the antioxidant, cytotoxic, and protective effects of three different quinazolinones in lipopolysaccharide murine microglia and hydrogen peroxide mouse neuroblastoma-2a cells [24]. Two quinazolinones with antioxidant activity (i.e., **17** and **18**, Figure 1) possessed an aromatic substituent with a hydroxyl group at position 2 of the main quinazolin-4(3*H*)-one ring. Herein, we decided to synthesize and investigate the antioxidant properties of two series of 2-substituted quinazolin-4(3*H*)-ones using three different antioxidant methods and compared them to the known structural analogs of phenolic antioxidants. Furthermore, their ability to chelate metal ions was also determined. Based on the results obtained, we gained some key information about the structure–antioxidant activity relationships of 2-substituted quinazolin-4(3*H*)-ones and defined quinazolinone with potent antioxidant activity and promising metal-chelating properties.

## 2. Results and Discussion

**Scheme 1:**
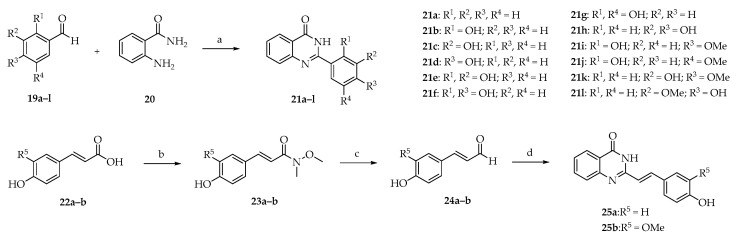
Reagents and conditions: (**a**) DMSO, 100 °C, 24–48 h; (**b**) CH_3_NHOCH_3_ × HCl, DMF, DIPEA, DMAP, EDC × HCl, rt, 24−72 h; (**c**) THF, DIBAL, −78 °C, 1 h; (**d**) anthranilamide (**20**), DMSO, 100 °C, 24 h.

The synthesis of 2-aryl-quinazolin-4(3*H*)-ones was performed according to the previously published procedures [25,26]. Briefly, in an open flask, the corresponding aldehydes **19a**–**l** reacted with antranilamide (**20**) in DMSO via aerobic oxidative cyclization to the corresponding quinazolinones **21a**–**l** (Scheme 1) [25,26]. The synthesis of (*E*)-2-(4-hydroxystyryl)quinazolin-4(3*H*)-one (**25a**) and (*E*)-2-(4-hydroxy-3-methoxystyryl)quinazolin-4(3*H*)-one (**25b**) have not yet been described. Thus, we decided to use derivatives of cinnamaldehyde and prepared the compounds **25a**–**b** using antranilamide (**20**) via the aforementioned procedure. (*E*)-3-(4-hydroxyphenyl)acrylaldehyde (**24a**) and (*E*)-3-(4-hydroxy-3-methoxyphenyl)acrylaldehyde (**24b**) were synthesized from the corresponding cinnamic acids **22a** and **22b** (i.e., *p*-coumaric and ferulic acids, respectively) [27]. Firstly, the coupling of a carboxylic group with *N,O*-dimethylhydroxylamine using 1-ethyl-3-(3-dimethylaminopropyl)carbodiimide (EDC) afforded Weinreb amides **23a**–**b**, which were further reduced with diisobutylaluminium hydride (DIBAL) to yield the cinnamaldehyde derivatives **24a** and **24b**, which reacted with antranilamide (**20**) in DMSO to form the final products **25a** and **25b**.

**Figure 2:**
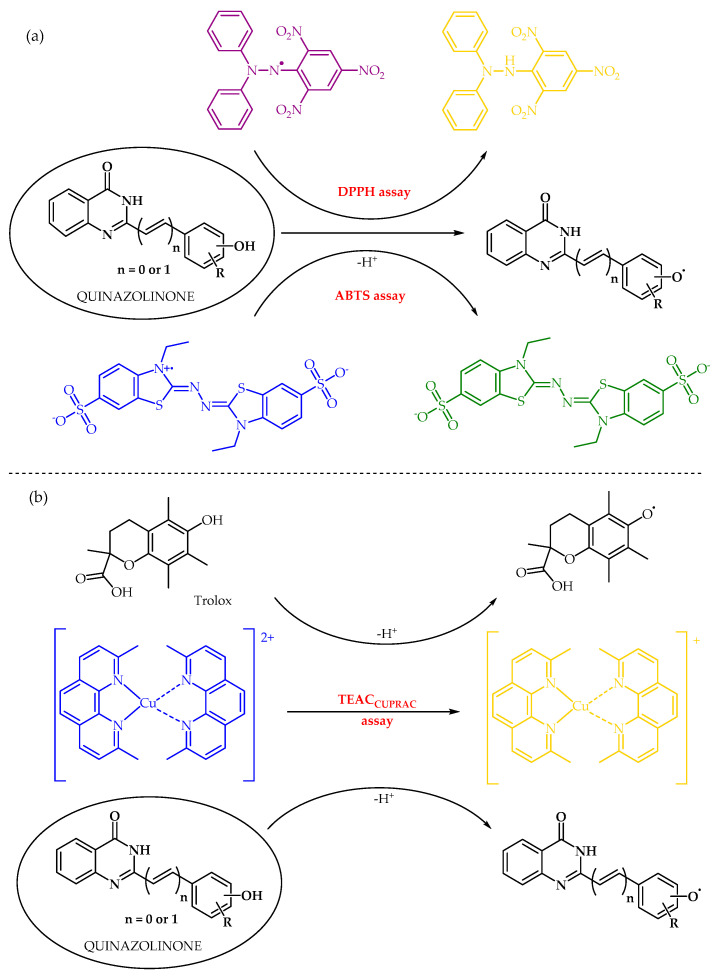
(**a**) Schematic presentation of DPPH and ABTS assay of quinazolin-4(3*H*)-ones; (**b**) schematic presentation of TEAC_CUPRAC_ assay of quinazolin-4(3*H*)-ones.

The antioxidant activity of synthesized quinazolin-4(3*H*)-ones **21a**–**l** and **25a**–**b** was firstly screened by one of the most commonly used methods, i.e., DPPH assay (Table 1, Figure 2a). The most potent radical scavenging activity was observed for three dihydroxy-substituted quinazolinones, i.e., **21e, 21g** and **21h** with EC_50_ values of 7.5, 7.4 and 7.2 μM, respectively. The second hydroxyl group needs to be in the ortho or para position, since the meta derivative (compound **21f**) loses most of its scavenging properties. This is in accordance with literature where increased antioxidant activity of phenolic compounds was reported if a second hydroxyl group is introduced in the ortho or para positions [28]. The majority of compounds with only one hydroxyl group did not show any activity with the exception of **21j**, **21l** and **25b**, which possessed additional methoxy substituent in the ortho or para position according to the hydroxyl group. It is known from the literature that the antioxidant activity of monophenols is significantly enhanced by one or two methoxy substituents in the ortho position relative to the hydroxyl group [28]. Comparing **21l** and **25b,** the only difference between them was the ethylene linker between the quinazolinone and benzene rings, which led to higher scavenging potency (approximately a 16.5-fold difference). Some compounds (such as **21b**, **21d** and **21f**) showed lower radical scavenging properties, as expected, according to their phenolic structure. Thus, we believe that the more appropriate assays for the determination of the antioxidant properties of 2-substituted quinazolin-4(3*H*)-ones are the ABTS and TEAC_CUPRAC_ assays (Table 1, Figure 2).

In the ABTS assay (Table 1) we were able to determine the EC_50_ values of monohydroxy derivatives in the range from 23.0 to 69.9 μM, with the meta derivative **21c** being the most potent. Among dihydroxy derivatives, there was no significant difference in potency (all EC_50_s were around 8 μM), while the EC_50_s for methoxy derivatives were in the range from 15.3 to 20.1 μM, with the most potent compounds **21k** and **21l** possessing the methoxy group in the ortho position. As mentioned previously, electron-donating groups (such as MeO) on the phenol ring significantly affected the antioxidant activity by decreasing the O−H bond dissociation enthalpy (BDE) of the phenol, leading to increased antioxidant activity [29]. However, the position of the methoxy group relative to the hydroxyl is important, since lower EC_50_ values (higher potency) were obtained in cases of ortho or para methoxy derivatives (**21k** and **21j**, respectively) compared to the meta derivative **21i**. This is in accordance with literature data where higher antioxidant properties were reported for natural and synthetic phenolic compounds with electron-donating groups in ortho or para positions [28,29,30]. The most potent compound was **25b** possessing an ethylene linker between quinazolinone and *ortho*-methoxyphenol moiety. The conjugated double bond also contributes to higher antioxidant properties due to the resonance stabilization effect on the phenoxyl radical [31], which was formed in the reaction between quinazolinone and ABTS (Figure 2).

In TEAC_CUPRAC_ assay (Table 1), compound **21e** exhibited the highest antioxidant capacity, with a TEAC value of 3.46. The other two dihydroxy derivatives, namely **21g** and **21h**, also showed good antioxidant properties (TEAC value of 2.62 and 2.74, respectively). Again, two hydroxyl groups in the ortho or para position according to each other, are the most optimal substitutions, since meta derivative **21f** showed much lower antioxidant capacity in the TEAC_CUPRAC_ assay. Among the methoxy derivatives, **21j** was the most potent antioxidant, whereas **25b** exhibited similar antioxidant properties to Trolox. The comparison of *p*-hydroxyphenyl derivative **21d** (TEAC value of 0.0315) and 4-hydroxystyryl derivative **25a** (TEAC value of 0.539) stresses the importance of the additional ethylene linker, leading to increased antioxidant potency due to the resonance stabilization of the formed phenoxyl radical. A similar pattern was also observed in the previously discussed ABTS assay.

**Figure 3:**
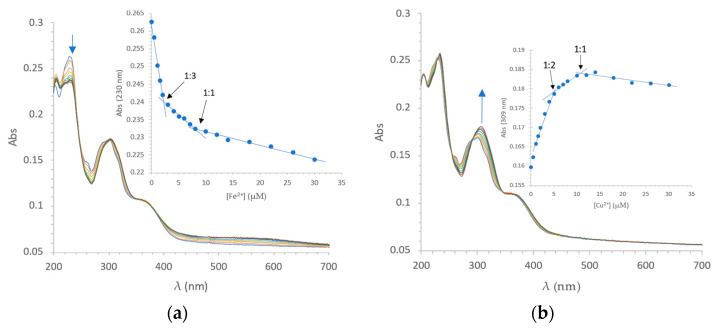
Spectrophotometric titration of 10 μM **21e** with (**a**) Fe^2+^ (0, 0.5, 1, 1.5, 2, 3, 4, 5, 6, 7, 8, 10, 12, 14, 18, 22, 26 and 30 μM) in 20 mM KPB buffer, pH 7.2. Inset, titration curve: absorbance change at 230 nm versus Fe^2+^. (**b**) Cu^2+^ (0, 0.5, 1, 1.5, 2, 3, 4, 5, 6, 7, 8, 10, 12, 14, 18, 22, 26 and 30 μM) in 20 mM KPB buffer, pH 7.2. Inset, titration curve: absorbance change at 309 nm versus Cu^2+^. Only the first nine absorption curves are shown.

In addition to three different antioxidant activity measurement assays ability of chelating metal ions (i.e., Fe^2+^ and Cu^2+^) of 2-substituted quinazolin-4(3*H*)-ones was also evaluated (Figure 3, Supplementary Materials, Figures S1–S14). Preliminary screening was performed by comparing the UV-Vis spectra of 10 mM solutions with spectra after the addition of 5, 10 and 20 mM Fe^2+^ or Cu^2+^ (Figures S1–S14). If the spectrum did not change shape and a slight dilution effect was seen, it was concluded that the compound does not bind metal ions. Two compounds, namely **21e** and **21h**, show pronounced chelation properties. Compound **21e** was selected for further study because of potent antioxidant activity (Table 1) and significant metal-chelating properties (Figure 3).

UV-Vis spectroscopic titration of **21e** with Fe^2+^ was performed in 20 mM KPB at pH 7.2. With the addition of Fe^2+^, the free **21e** absorption (λ_max_ = 230 nm) rapidly decreased and the newly formed Fe^2+^-complex band red shifted around 300 nm. The presence of a clear isosbestic point (λ_max_ = 301 nm) suggested the formation of the **21e**-Fe^2+^ complex (Figure 3a). A plot of **21e**-Fe^2+^ complex absorption at 230 nm against the Fe^2+^ concentration is displayed in Figure 3a. The titration curve displays the formation of 1:3 and 1:1 Fe^2+^: **21e** complexes.

Similarly, complex formation between **21e** and Cu^2+^ was studied. The addition of Cu^2+^ to **21e** produced a new band at 309 nm, of higher intensity than the 301 nm band of **21e**-Fe^2+^. The Cu^2+^ was partially reduced to Cu^+^, and the catechol was oxidised to the orthoquinone derivative of **21e** (Figure S16). A plot of the **21e**-Cu^2+^ complex absorption at 309 nm against the Cu concentration is presented in Figure 3b. The titration curve displays the formation of 1:2 and 1:1 Cu: **21e** complexes, which was confirmed by ESI-high resolution mass spectrometry measurements (Figure S15).

## 3. Materials and Methods

### 3.1. Chemistry

The reagents and solvents were obtained from commercial sources (Sigma-Aldrich, Acros Organics, Alfa Aesar, TCI, Merck) and used without further purification. Thin-layer chromatography (TLC) on silica gel plates (Merck DC Fertigplatten Kieselgel 60 GF254) was used to monitor the reaction. TLC spots were visualized under UV light and/or stained with the appropriate dyeing agents (Iron(III) chloride, 2,4-dinitrophenylhydrazine, bromocresol green). Flash column chromatography was performed on Merck silica gel 60 (mesh size, 70–230). Yields refer to the purified products and were not optimized. The ^1^H and ^13^C NMR spectra were recorded at 295 K in DMSO-*d*_6_ on a Bruker Avance III NMR spectrometer equipped with a broadband decoupling inverse 1H probe. The coupling constants (*J*) were in Hz, and the splitting patterns were designated as: s, singlet; br s, broad singlet; d, doublet; dd, double doublet; t, triplet; dt, double triplet; ddd, double of doublet of doublet; and m, multiplet. The mass spectra and high-resolution mass measurements were performed at the Faculty of Pharmacy, University of Ljubljana, on an ADVION Expression CMSL mass spectrometer (Advion Inc., New York, NY, USA) and an Exactive ^TM^ Plus Orbitrap mass spectrometer (Thermo Fischer Scientific Inc., Waltham, MA, USA), respectively.

#### General Procedure for the Synthesis of Quinazolinones **21a**–**l**

Quinazolinones **21a**–**l** were synthesized according to the previously reported procedures, with some modifications [25]. Appropriate aldehyde **19a**–**l** (1.2 equiv.) and anthranilamide (**20**) (1.0 equiv.) were dissolved in DMSO (5 mL). The reaction mixture was stirred at 100 °C in an open flask for 24–48 h and cooled to room temperature. Up to 100 mL of water was added to form the precipitate, which was collected by filtration and washed with water and methanol. If the product was not pure according to thin-layer chromatography, it was further recrystallized from ethanol.

2-phenylquinazolin-4(3*H*)-one (**21a**): synthesized from benzaldehyde **19a** (1.2 mmol, 0.122 mL) and anthranilamide **20** (1.0 mmol, 0.136 g). Yield 81%; white crystals. Rf = 0.76 (DCM/MeOH = 15/1 *v*/*v*); ^1^H NMR (400 MHz, DMSO-*d*_6_): δ (ppm) = 7.52–7.61 (m, 4H), 7.75–7.77 (m, 1H), 7.83–7.87 (m, 1H), 8.16–8.21 (m, 3H), 12.57 (s, 1H); ^13^C NMR (100 MHz, DMSO-*d*_6_): δ (ppm) = 120.93, 125.84, 126.61, 127.49, 127.74, 128.61, 131.4, 132.67, 134.63, 148.70, 152.31, 162.24; HRMS (ESI+) *m/z* calc. for C_14_H_11_N_2_O [M + H]^+^ 223.08659, found 223.08642; IR (ATR): ν cm^−1^ = 3063, 1661, 1599, 1557, 1474, 1336, 1290, 1190, 1143, 1102, 1024, 940, 822, 765, 687, 617, 536.
2-(2-hydroxyphenyl)quinazolin-4(3*H*)-one (**21b**): synthesized from salicylaldehyde **19b** (1.2 mmol, 0.126 mL) and anthranilamide **20** (1.0 mmol, 0.136 g). Yield 74%; yellow-white crystals. Rf = 0.52 (EtOAc/*n*-hexane = 1/1 *v*/*v*); ^1^H NMR (400 MHz, DMSO-*d*_6_): δ (ppm) = 6.95–7.03 (m, 2H), 7.44–7.58 (m, 2H), 7.77–7.90 (m, 2H), 8.16–8.25 (m, 2H); 12.49 (s, 1H), 13.81 (s, 1H); ^13^C NMR (100 MHz, DMSO-*d*_6_): δ (ppm) = 113.71, 117.87, 118.81, 120.73, 126.03, 126.96, 127.68, 133.71, 135.02, 146.11, 153.69, 160.02, 161.37; HRMS (ESI+) *m/z* calc. for C_14_H_11_N_2_O_2_ [M + H]^+^ 239.08150, found 239.08085; IR (ATR): ν cm^−1^ = 3099, 1666, 1604, 1559, 1511, 1491, 1460, 1438, 1396, 1330, 1299, 1251, 1226, 1166, 1146, 1124, 1068, 1040, 1020, 951, 874, 825, 793, 760, 686.
2-(3-hydroxyphenyl)quinazolin-4(3*H*)-one (**21c**): synthesized from 3-hydroxybenzaldehyde **19c** (1.2 mmol, 0.146 g) and anthranilamide **20** (1.0 mmol, 0.136 g). Yield 67%; yellow-white crystals. Rf = 0.18 (EtOAc/*n*-hexane = 1/1 *v*/*v*); ^1^H NMR (400 MHz, DMSO-*d*_6_): δ (ppm) = 6.98–7.00 (m, 1H), 7.33–7.37 (m, 1H), 7.50–7.54 (m, 1H), 7.60–7.62 (m, 2H), 7.72–7.74 (m, 1H), 7.82–7.86 (m, 1H), 8.15–8.17 (m, 1H), 9.79 (s, 1H), 12.46 (s, 1H); ^13^C NMR (100 MHz, DMSO-*d*_6_): δ (ppm) = 114.55, 118.32, 118.49, 120.98, 125.83, 126.51, 127.44, 129.66, 134.01, 134.58, 148.70, 152.32, 157.49, 162.17; HRMS (ESI+) *m/z* calc. for C_14_H_11_N_2_O_2_ [M + H]^+^ 239.08150, found 239.08067; IR (ATR): ν cm^−1^ = 3193, 3067, 1657, 1604, 1560, 1510, 1468, 1443, 1370, 1337, 1298, 1245, 1214, 1138, 1091, 1019, 998, 972, 886, 823, 802, 770, 719, 671.
2-(4-hydroxyphenyl)quinazolin-4(3*H*)-one (**21d**): synthesized from 4-hydroxybenzaldehyde **19d** (1.2 mmol, 0.146 g) and anthranilamide **20** (1.0 mmol, 0.136 g). Yield 50%; white solid. Rf = 0.34 (EtOAc/*n*-hexane = 2/1 *v*/*v*); ^1^H NMR (400 MHz, DMSO-*d*_6_): δ (ppm) = 6.89 (d, *J* = 8.6 Hz, 2H), 7.46 (ddd, *J* = 7.8, 7.1, 1.2 Hz, 1H), 7.68 (d, *J* = 7.6 Hz, 1H), 7.79 (ddd, *J* = 8.3, 6.9, 1.4 Hz, 1H), 8.07–8.13 (m, 3H), 10.17 (s, 1H), 12.32 (s, 1H); ^13^C NMR (100 MHz, DMSO-*d*_6_): δ (ppm) = 115.32, 120.54, 123.17, 125.78, 125.89, 127.16, 129.55, 134.48, 149.01, 152.08, 160.51, 162.29; HRMS (ESI+) *m/z* calc. for C_14_H_11_N_2_O_2_ [M + H]^+^ 239.0821, found 239.0824; IR (ATR): ν cm^−1^ = 3183, 3069, 2915, 2594, 1656, 1602, 1577, 1556, 1520, 1488, 1451, 1431, 1376, 1344, 1324, 1310, 1286, 1258, 1233, 1182, 1150, 1107, 1082, 1027.
2-(2,3-dihydroxyphenyl)quinazolin-4(3*H*)-one (**21e**): synthesized from 2,3-dihydroxybenzaldehyde **19e** (1.2 mmol, 0.166 g) and anthranilamide **20** (1.0 mmol, 0.136 g). Yield 71%; yellow crystals. Rf = 0.25 (DCM/MeOH = 15/1 *v*/*v*); ^1^H NMR (400 MHz, DMSO-*d*_6_): δ (ppm) = 6.76–6.80 (m, 1H), 6.98–7.00 (m, 1H), 7.52–7.57 (m, 1H), 7.70–7.76 (m, 2H), 7.85–7.89 (m, 1H), 8.15–8.17 (m, 1H), 9.24 (s, 1H), 12.44 (s, 1H), 14.01 (s, 1H); ^13^C NMR (100 MHz, DMSO-*d*_6_): δ (ppm) = 113.99, 117.90, 118.77, 119.38, 121.14, 126.30, 126.57, 127.41, 135.55, 146.42, 146.99, 149.89, 154.70, 161.87; HRMS (ESI+) *m/z* calc. for C_14_H_11_N_2_O_3_ [M + H]^+^ 255.07642, found 255.07582; IR (ATR): ν cm^−1^ = 3435, 3107, 1667, 1612, 1584, 1570, 1508, 1445, 1370, 1332, 1280, 1214, 1176, 1145, 1076, 1002, 915, 815, 769, 731, 626, 548, 533.
2-(2,4-dihydroxyphenyl)quinazolin-4(3*H*)-one (**21f**): synthesized from 2,4-dihydroxybenzaldehyde **19f** (1.2 mmol, 0.166 m) and anthranilamide **20** (1.0 mmol, 0.136 g). Yield 45%; yellow-white crystals. Rf = 0.30 (DCM/MeOH = 15/1 *v*/*v*); ^1^H NMR (400 MHz, DMSO-*d*_6_): δ (ppm) = 6.35–6.40 (m, 2H), 7.46–7.50 (m, 1H), 7.66–7.68 (m, 1H), 7.80–7.84 (m, 1H), 8.10–8.13 (m, 2H), 10.28 (s, 1H), 12.27 (s, 1H), 14.24 (s, 1H); ^13^C NMR (100 MHz, DMSO-*d*_6_): δ (ppm) = 103.85, 105.50, 108.10, 120.66, 125.91, 126.52, 126.67, 129.53, 135.44, 146.69, 154.46, 161.93, 163.05, 163.13; HRMS (ESI+) *m/z* calc. for C_14_H_11_N_2_O_3_ [M + H]^+^ 255.07642, found 255.07610; IR (ATR): ν cm^−1^ = 3195, 1670, 1605, 1525, 1441, 1334, 1287, 1230, 1179, 1148, 1067, 1018, 979, 949, 823, 761, 684, 622.
2-(2,5-dihydroxyphenyl)quinazolin-4(3*H*)-one (**21g**): synthesized from 2,5-dihydroxybenzaldehyde **19g** (1.2 mmol, 0.166 g) and anthranilamide **20** (1.0 mmol, 0.136 g). Yield 56%; brown crystals. Rf = 0.26 (DCM/MeOH = 15/1 *v*/*v*); ^1^H NMR (400 MHz, DMSO-*d*_6_): δ (ppm) = 6.85–6.95 (m, 2H), 7.51–7.56 (m, 1H), 7.63–7.63 (m, 1H), 7.73–7.75 (m, 1H), 7.83–7.87 (m, 1H), 8.14–8.16 (m, 1H), 9.12 (s, 1H), 12.32 (s, 1H), 12.65 (s, 1H); ^13^C NMR (100 MHz, DMSO-*d*_6_): δ (ppm) = 113.88, 114.88, 118.80, 121.18, 121.99, 126.46, 126.77, 127.22, 135.38, 147.23, 150.08, 152.63, 153.78, 161.76; HRMS (ESI+) *m/z* calc. for C_14_H_11_N_2_O_3_ [M + H]^+^ 255.07642, found 255.07617; IR (ATR): ν cm^−1^ = 3198, 3093, 1609, 1561, 1509, 1480, 1368, 1326, 1302, 1251, 1200, 1124, 979, 917, 873, 816, 767, 678, 621, 526.
2-(3,4-dihydroxyphenyl)quinazolin-4(3*H*)-one (**21h**): synthesized from 3,4-dihydroxybenzaldehyde **19h** (1.2 mmol, 0.166 g) and anthranilamide **20** (1.0 mmol, 0.136 g). Yield 68%; yellow-white crystals. Rf = 0.16 (DCM/MeOH = 15/1 *v*/*v*); ^1^H NMR (400 MHz, DMSO-*d*_6_): δ (ppm) = 6.84–6.86 (m, 1H), 7.44–7.48 (m, 1H), 7.55–7.58 (m, 1H), 7.66–7.70 (m, 2H), 7.78–7.80 (m, 1H), 8.11–8.13 (m, 1H), 9.34 (s, 1H), 9.69 (s, 1H), 12.25 (s, 1H); ^13^C NMR (100 MHz, DMSO-*d*_6_): δ (ppm) = 115.64, 115.74, 120.07, 121.07, 124.08, 126.30, 126.34, 127.61, 134.97, 145.84, 149.52 (2C), 152.70, 162.76; HRMS (ESI+) *m/z* calc. for C_14_H_11_N_2_O_3_ [M + H]^+^ 255.07642, found 255.07608; IR (ATR): ν cm^−1^ = 3455, 3035, 1643, 1602, 1528, 1468, 1403, 1290, 1249, 1199, 1149, 1116, 1078, 978, 859, 770, 686, 645, 585, 525.
2-(2-hydroxy-4-methoxyphenyl)quinazolin-4(3*H*)-one (**21i**): synthesized from 2-hydroxy-4-methoxybenzaldehyde **19i** (1.2 mmol, 0.183 g) and anthranilamide **20** (1.0 mmol, 0.136 g). Yield 83%; light orange crystals. Rf = 0.24 (EtOAc/*n*-hexane = 1/1 *v*/*v*); ^1^H NMR (400 MHz, DMSO-*d*_6_): δ (ppm) = 3.81 (s, 3H), 6.52–6.56 (m, 2H), 7.48–7.52 (m, 1H), 7.69–7.71 (m, 1H), 7.81–7.85 (m, 1H), 8.12–8.21 (m, 2H), 12.37 (s, 1H), 14.42 (s, 1H); ^13^C NMR (100 MHz, DMSO-*d*_6_): δ (ppm) = 55.45, 101.74, 106.11, 106.48, 120.32, 125.48, 126.02, 126.42, 128.76, 135.00, 145.69, 153.79, 161.40, 162.70, 163.67; HRMS (ESI+) *m/z* calc. for C_15_H_13_N_2_O_3_ [M + H]^+^ 269.09207, found 269.09146; IR (ATR): ν cm^−1^ = 3077, 1670, 1605, 1562, 1525, 1501, 1464, 1405, 1337, 1252, 1211, 1182, 1155, 1131, 1069, 1032, 966, 945, 859, 818, 763, 689.
2-(2-hydroxy-5-methoxyphenyl)quinazolin-4(3*H*)-one (**21j**): synthesized from 2-hydroxy-5-methoxybenzaldehyde **19j** (1.2 mmol, 0.183 g) and anthranilamide **20** (1.0 mmol, 0.136 g). Yield 71%; dark yellow crystals. Rf = 0.49 (EtOAc/*n*-hexane = 1/1 *v*/*v*); ^1^H NMR (400 MHz, DMSO-*d*_6_): δ (ppm) = 3.81 (s, 3H), 6.93–6.95 (m, 1H), 7.06–7.09 (m, 1H), 7.53–7.57 (m, 1H), 7.75–7.78 (m, 2H), 7.84–7.88 (m, 1H), 8.15–8.17 (m, 1H), 12.58 (s, 1H), 13.48 (s, 1H); ^13^C NMR (100 MHz, DMSO-*d*_6_): δ (ppm) = 55.82, 110.11, 112.97, 118.93, 120.65, 121.78, 126.01, 126.94, 135.02, 146.08, 151.69, 153.62, 154.40, 161.48; HRMS (ESI+) *m/z* calc. for C_15_H_13_N_2_O_3_ [M + H]^+^ 269.09207, found 269.09125; IR (ATR): ν cm^−1^ = 3094, 2989, 2837, 1660, 1611, 1562, 1508, 1487, 1456, 1426, 1392, 1332, 1294, 1253, 1223, 1144, 1046, 967, 912, 878, 853, 833, 813, 764, 677.
2-(3-hydroxy-4-methoxyphenyl)quinazolin-4(3*H*)-one (**21k**): synthesized from 3-hydroxy-4-methoxybenzaldehyde **19k** (1.1 mmol, 0.167 g) and anthranilamide **20** (1.0 mmol, 0.136 g). Yield 51%; light yellow crystals. Rf = 0.12 (EtOAc/*n*-hexane = 1/1 *v*/*v*); ^1^H NMR (400 MHz, DMSO-*d*_6_): δ (ppm) = 3.87 (s, 3H), 7.05–6.08 (m, 1H), 7.47–7.51 (m, 1H), 7.68–7.72 (m, 3H), 7.80–7.84 (m, 1H), 8.12–8.14 (m, 1H), 9.40 (s, 1H), 12.33 (s, 1H); ^13^C NMR (100 MHz, DMSO-*d*_6_): δ (ppm) = 111.45, 114.73, 119.33, 120.62, 124.89, 125.83, 126.09, 127.01, 134.54, 146.431, 148.68, 150.73, 152.07, 162.30; HRMS (ESI+) *m/z* calc. for C_15_H_13_N_2_O_3_ [M + H]^+^ 269.09207, found 269.09118; IR (ATR): ν cm^−1^ = 3532, 2974, 1652, 1603, 1577, 1514, 1490, 1437, 1344, 1292, 1256, 1215, 1196, 1142, 1107, 1075, 1021, 925, 887, 864, 832, 734, 712, 689.
2-(4-hydroxy-3-methoxyphenyl)quinazolin-4(3*H*)-one (**21l**): synthesized from 4-hydroxy-3-methoxybenzaldehyde **19l** (1.1 mmol, 0.167 g) and anthranilamide **20** (1.0 mmol, 0.136 g). Yield 71%; yellow-white solid. Rf = 0.30 (EtOAc/*n*-hexane = 1/1 *v*/*v*); ^1^H NMR (400 MHz, DMSO-*d*_6_): δ (ppm) = 3.90 (s, 3H), 6.92 (d, *J* = 8.3 Hz, 1H), 7.44–7.48 (m, 1H), 7.69 (d, *J* = 7.9 Hz, 1H), 7.74–7.79 (m, 2H), 7.80 (d, *J* = 1.8 Hz, 1H), 8.12 (dd, *J* = 7.8, 0.8 Hz, 1H) 9.78 (s, 1H), 12.36 (s, 1H); ^13^C NMR (100 MHz, DMSO-*d*_6_): δ (ppm) = 55.83, 111.37, 115.47, 120.64, 121.55, 123.45, 125.88, 126.00, 127.28, 134.57, 147.54, 149.07, 150.00, 152.09, 162.44; HRMS (ESI-) *m/z* calc. for C_15_H_11_N_2_O_3_ [M − H]^−^ 267.07752, found 267.07741; IR (ATR): ν cm^−1^ = 3488, 3170, 3128, 3087, 3012, 2972, 2944, 2847, 1659, 1610, 1573, 1522, 1481, 1458, 1444, 1344, 1284, 1247, 1211, 1173, 1147, 1119, 1072, 1027, 1018, 966, 895, 864, 820, 765.

Quinazolinones **25a**–**b** were synthesized from cinnamic acids **22a**–**b** according to the previously reported procedures, with some modifications [25,27]:

(*E*)-3-(4-hydroxyphenyl)-*N*-methoxy-*N*-methylacrylamide (**23a**): *N*,*O*-dimethylhydroxylamine hydrochloride (2.93 g, 30.0 mmol) was suspended in *N*,*N*-dimethylformamide (10 mL). After the addition of *N*,*N*-diisopropylethylamine (4.53 mL, 26.0 mmol) and 4-(dimethylamino)pyridine (0.367 g, 3.0 mmol), *p*-coumaric acid **22a** (3.28 g, 20.0 mmol) and *N*-(3-dimethylaminopropyl)-*N′*-ethylcarbodiimide hydrochloride (3.93 g, 20.5 mmol) were added. The reaction mixture was stirred at room temperature for 24 h. Then, ethyl acetate (300 mL) was added and the obtained solution was washed with 1 M hydrochloric acid (2 × 100 mL), saturated sodium hydrogen carbonate solution (2 × 100 mL) and brine (100 mL). The layers were separated, and the organic phase was dried over anhydrous sodium sulfate, filtered and concentrated in vacuo to obtain (*E*)-3-(4-hydroxyphenyl)-*N*-methoxy-*N*-methylacrylamide (**23a**) as a white solid [27] in 80% yield. Rf = 0.44 (EtOAc/n-hexane = 2/1 *v*/*v*); ^1^H NMR (400 MHz, DMSO-*d*_6_): δ (ppm) = 3.18 (s, 3H), 3.72 (s, 3H), 6.79–6.82 (m, 2H), 6.89 (d, *J* = 15.7 Hz, 1H), 7.48 (d, *J* = 15.7 Hz, 1H), 7.52–7.55 (m, 2H), 9.94 (s, 1H); HRMS (ESI-) *m/z* calc. for C_11_H_12_NO_3_ [M − H]^−^ 206.08227, found 206.08187; IR (ATR): ν cm^−1^ = 3078, 3006, 2941, 2812, 2690, 2621, 1638, 1569, 1509, 1439, 1384, 1279, 1267, 1236, 1205, 1164, 1144, 1098, 1027, 1000, 975, 942, 826, 766.
(*E*)-3-(4-hydroxy-3-methoxyphenyl)-*N*-methoxy-*N*-methylacrylamide (**23b**): *N*,*O*-dimethylhydroxylamine hydrochloride (2.93 g, 30.0 mmol) was suspended in *N*,*N*-dimethylformamide (10 mL). After the addition of *N*,*N*-diisopropylethylamine (4.53 mL, 26.0 mmol) and 4-(dimethylamino)pyridine (0.367 g, 3.0 mmol), *p*-ferulic acid **22b** (3.88 g, 20.0 mmol) and *N*-(3-dimethylaminopropyl)-*N′*-ethylcarbodiimide hydrochloride (3.93 g, 20.5 mmol) were added. The reaction mixture was stirred at room temperature for 72 h. Then, ethyl acetate (300 mL) was added, and the obtained solution was washed with 1 M hydrochloric acid (2 × 100 mL), saturated sodium hydrogen carbonate solution (2 × 100 mL) and brine (100 mL). The layers were separated, and the organic phase was dried over anhydrous sodium sulfate, then filtered and concentrated in vacuo. The crude product was purified over a short column of silica gel (EtOAc/n-hexane = 2/1 as an eluent) to obtain (*E*)-3-(4-hydroxy-3-methoxyphenyl)-*N*-methoxy-*N*-methylacrylamide (**23b**) as a white solid [27] in 77% yield. Rf = 0.35 (EtOAc/n-hexane = 2/1 *v*/*v*); ^1^H NMR (400 MHz, DMSO-*d*_6_): δ (ppm) = 3.19 (s, 3H), 3.73 (s, 3H), 3.83 (s, 3H), 6.81 (d, *J* = 8.1 Hz, 1H), 6.92 (d, *J* = 15.7 Hz, 1H), 7.14 (dd, *J* = 8.2, 1.9 Hz, 1H), 7.26 (d, *J* = 1.9 Hz, 1H), 7.50 (d, *J* = 15.7 Hz, 1H), 9.52 (s, 1H); HRMS (ESI-) *m/z* calc. for C_12_H_14_NO_4_ [M − H]^−^ 236.09283, found 236.09269; IR (ATR): ν cm^−1^ = 3288, 3007, 2940, 2835, 1647, 1610, 1597, 1587, 1509, 1465, 1452, 1427, 1382, 1279, 1265, 1230, 1200, 1160, 1148, 1120, 1097, 1034, 999, 979, 952, 835, 805, 729.
(*E*)-3-(4-hydroxyphenyl)acrylaldehyde (**24a**): The solution of (*E*)-3-(4-hydroxyphenyl)-*N*-methoxy-*N*-methylacrylamide **23a** (1.243 g, 6.0 mmol) in anhydrous tetrahydrofuran (50 mL) was cooled to −78 °C. 1 M solution of diisobutylaluminium hydride in hexane (12.0 mL, 12.0 mmol), previously cooled to −78 °C, was added dropwise to the reaction mixture via a cannula. The solution was stirred for an hour at −78 °C before excess DIBAL was quenched by dropwise addition of ethyl acetate (5 mL). The solution was stirred for additional half an hour and the cooling bath was then removed. Ethyl acetate (150 mL) was added to the resulting reaction mixture, which was then washed with 1 M hydrochloric acid (3 × 50 mL) and brine (50 mL). The organic phase was dried over anhydrous sodium sulfate and concentrated in vacuo to obtain crude *(E*)-3-(4-hydroxyphenyl)acrylaldehyde **24a** (0.741 g) as a yellow solid [27], which was used in the next reaction without further purification. Rf = 0.69 (EtOAc/n-hexane = 2/1 *v*/*v*); ^1^H NMR (400 MHz, DMSO-*d*_6_): δ (ppm) = 6.66 (dd, *J* = 15.8, 7.9 Hz, 1H), 6.82–6.86 (m, 2H), 7.58–7.61 (m, 2H), 7.61 (d, *J* = 15.8 Hz, 1H), 9.58 (d, *J* = 7.9 Hz, 1H), 10.21 (s, 1H); HRMS (ESI+) *m/z* calc. for C_9_H_9_O_2_ [M + H]^+^ 149.05971, found 149.05972; IR (ATR): ν cm^−1^ = 3078, 2824, 1638, 1598, 1573, 1510, 1460, 1383, 1323, 1282, 1236, 1205, 1170, 1137, 1105, 1001, 971, 815, 759.
(*E*)-3-(4-hydroxy-3-methoxyphenyl)acrylaldehyde (**24b**): The solution of (*E*)-3-(4-hydroxy-3-methoxyphenyl)-*N*-methoxy-*N*-methylacrylamide **23b** (1.424 g, 6.0 mmol) in anhydrous tetrahydrofuran (50 mL) was cooled to −78 °C. 1 M solution of diisobutylaluminium hydride in hexane (12.0 mL, 12.0 mmol), previously cooled to −78 °C, was added dropwise to the reaction mixture via a cannula. The solution was stirred for an hour at −78 °C before excess DIBAL was quenched by dropwise addition of ethyl acetate (5 mL). The solution was stirred for an additional half an hour and the cooling bath was then removed. Ethyl acetate (150 mL) was added to the resulting reaction mixture, which was then washed with 1 M hydrochloric acid (3 × 50 mL) and brine (50 mL). The organic phase was dried over anhydrous sodium sulfate and concentrated in vacuo to obtain crude (*E*)-3-(4-hydroxy-3-methoxyphenyl)acrylaldehyde **24b** (0.909 g) as a yellow solid [27], which was used in the next reaction without further purification. Rf = 0.65 (EtOAc/*n*-hexane = 2/1 *v*/*v*); ^1^H NMR (400 MHz, DMSO-*d*_6_): δ (ppm) = 3.82 (s, 3H), 6.74 (dd, *J* = 15.7, 7.9 Hz, 1H), 6.84 (d, *J* = 8.2 Hz, 1H), 7.17 (dd, *J* = 8.2, 1.9 Hz, 1H), 7.34 (d, *J* = 1.9 Hz, 1H), 7.59 (d, *J* = 15.7 Hz, 1H), 9.58 (d, *J* = 7.9 Hz, 1H), 9.82 (s, 1H); HRMS (ESI+) *m/z* calc. for C_10_H_11_O_3_ [M + H]^+^ 179.07027, found 179.07006; IR (ATR): ν cm^−1^ = 3351, 2982, 2840, 2736, 1730, 1660, 1621, 1583, 1510, 1464, 1430, 1373, 1282, 1203, 1161, 1116, 1028, 969, 809, 760, 743.
(*E*)-2-(4-hydroxystyryl)quinazolin-4(3*H*)-one (**25a**): To a solution of (*E*)-3-(4-hydroxyphenyl)acrylaldehyde **24a** (0.741 g, 5.0 mmol) in DMSO (20 mL), anthranilamide **20** (0.681 g, 5.0 mmol) was added and stirred at 100 °C in an open flask for 24 h. After cooling to room temperature, 20 mL of water was added to form the precipitate, which filtered off and recrystallized from ethanol. The solid was suspended in 5 mL of acetone and filtered off to obtain pure (*E*)-2-(4-hydroxystyryl)quinazolin-4(3*H*)-one (**25a**) as a yellow solid in 16% yield. Rf = 0.54 (EtOAc/*n*-hexane = 2/1 *v*/*v*); ^1^H NMR (400 MHz, DMSO-*d*_6_): δ (ppm) = 6.78 (d, *J* = 16.1 Hz, 1H), 6.82–6.86 (m, 2H), 7.42−7.47 (m, 1H), 7.48–7.52 (m, 2H), 7.64 (d, *J* = 7.9 Hz, 1H), 7.78 (d, *J* = 8.5, 7.2, 1.6 Hz, 1H), 7.87 (d, *J* = 16.1 Hz, 1H), 8.09 (dd, *J* = 7.9, 1.2 Hz, 1H), 9.97 (1H), 12.23 (s, 1H); ^13^C NMR (100 MHz, DMSO-*d*_6_): δ (ppm) = 116.00, 117.36, 120.93, 125.86, 125.89, 126.11, 126.99, 129.54, 134.48, 138.58, 149.26, 151.95, 159.33, 161.81; HRMS (ESI+) *m/z* calc. for C_16_H_13_N_2_O_2_ [M + H]^+^ 265.09715 found 265.09653; IR (ATR): ν cm^−1^ = 3191, 3144, 2934, 2585, 1667, 1645, 1601, 1577, 1558, 1515, 1470, 1440, 1364, 1342, 1322, 1309, 1285, 1270, 1251, 1209, 1170, 1141, 1102, 1009, 970, 818, 766.
(*E*)-2-(4-hydroxy-3-methoxystyryl)quinazolin-4(3*H*)-one (**25b**): To a solution of *(E*)-3-(4-hydroxyphenyl)acrylaldehyde **24b** (0.891 g, 5.0 mmol) in DMSO (20 mL), anthranilamide 20 (0.681 g, 5.0 mmol) was added and stirred at 100 °C in an open flask for 24 h. After cooling to room temperature, 30 mL of water was added to form the precipitate, which was recrystallize from ethanol to obtain pure (*E*)-2-(4-hydroxy-3-methoxystyryl)quinazolin-4(3*H*)-one (**25b**) as a yellow solid in 30% yield. Rf = 0.25 (EtOAc/*n*-hexane = 2/1 *v*/*v*); ^1^H NMR (400 MHz, DMSO-*d*_6_): δ (ppm) = 3.84 (s, 3H), 6.81–6.85 (m, 2H), 7.09 (dd, *J* = 8.1, 1.2 Hz, 1H), 7.25 (d, *J* = 1.2 Hz, 1H), 7.42–7.47 (m, 1H), 7.63 (d, *J* = 8.1 Hz, 1H), 7.76–7.81 (m, 1H), 7.87 (d, *J* = 16.1 Hz, 1H), 8.09 (dd, *J* = 7.9, 1.6 Hz, 1H), 9.58 (1H), 12.19 (s, 1H); ^13^C NMR (100 MHz, DMSO-*d*_6_): δ (ppm) = 55.62, 110.87, 115.82, 117.69, 120.92, 121.94, 125.88, 126.60, 126.96, 134.49, 138.84, 148.00, 148.79, 149.26, 151.95, 161.81; HRMS (ESI+) *m/z* calc. for C_17_H_15_N_2_O_3_ [M + H]^+^ 295.10772, found 295.10715; IR (ATR): ν cm^−1^ = 3184, 3049, 2987, 2933, 2832, 1679, 1644, 1601, 1573, 1560, 1518, 1469, 1447, 1425, 1345, 1326, 1279, 1257, 1244, 1203, 1164, 1119, 1038, 1005, 960, 886, 834, 758.

### 3.2. Antioxidant Activity Determined by DPPH^•^, ABTS and CUPRAC Assays

Free-radical scavenging activity was evaluated employing the DPPH^•^ and ABTS assays. In our previous study [32], we found that polyphenols are slow-reacting antioxidants; thus, we extended the incubation period in the DPPH and ABTS assays to 90 min. The 2,2-Diphenyl-1-picrylhydrazyl radical (DPPH^•^) was dissolved in methanol (150 µL, 140 µM) and added to 150 µL methanol solution of the tested compound (62.5–500 µM), methanol (negative control) or α-tocopherol (positive control) in each well of a flat-bottomed 96-well microliter plate (TPP, Tissue Culture Test Plates). The reaction between DPPH^•^ and the tested compound was then monitored at λ = 517 nm using a Synergy H4 Hybrid Multi-Mode Microplate Reader (Bio-Tek Instruments, Inc) at t = 20 °C in the dark after 90 min. Each set of experiments was performed in triplicate.

For the ABTS assay, a slightly modified procedure described in the literature [33] was used. To 10 mL of 7 mM stock solution of 2,2′-azinobis(3-ethylbenzothiazoline-6-sulfonic acid ammonium salt) (ABTS) was added 178 µL 140 mM solution of potassium persulfate. Working solution was allowed to react for 16 h at room temperature in the dark. The solution was than diluted by mixing 1 mL ABTS^•+^ with 32 mL of ethanol (96%) to obtain an absorbance of 1.1 units at 734 nm after mixing with the same volume of ethanol. Solutions of tested compounds and solution of a standard (Trolox) were freshly prepared in 96% ethanol at 1 mM concentration. ABTS^•+^ solution (150 µL, 215 µM) was added to 150 µL ethanol solution of the tested compound (2.5–100 µM) or ethanol (negative control) in each well of a flat-bottomed 96-well microliter plate (TPP, Tissue Culture Test Plates). The reaction between ABTS^•+^ and tested compound was then monitored at λ = 734 nm by using a Synergy H4 Hybrid Multi-Mode Microplate Reader (Bio-Tek Instruments, Inc) at T = 20 °C in the dark after 90 min. Each set of experiments was performed in triplicate.

Trolox equivalent antioxidant capacity (TEAC_CUPRAC_) of compounds (**21a**–**l**, **25a**–**b**) was determined using its Cu^2+^ reducing capability in the presence on neocuproine by the CUPRAC method [34]. Solution compounds (**21a**–**l**, **25a**–**b**) and the solution of the standard (Trolox) were freshly prepared in 96% ethanol at 1 mM concentration. To a test tube, 1 mL each of CuCl_2_ (10 mM in water), neocuproine (7.5 mM in 96% ethanol) and ammonium acetate buffer (pH 7, 1 mM in water) solutions were added. Compound (or standard) solution (x mL) and water (1.10 − x) mL were added to the mixture to obtain the final volume 4.1 mL. The tubes were closed by parafilm, and the mixtures were vortexed and incubated for 60 min at room temperature. Absorbance at 450 nm was recorded against a reagent blank using the UV-Vis spectrophotometer (Agilent Cary 3500 UV-Vis spectrophotometer with the Compact Peltier UV-Vis Module). The molar absorptivity (ε) for each antioxidant was calculated from the slope of the calibration line by plotting absorbance versus concentration (the calibration curve obtained can be found in the Supplementary material). TEAC_CUPRAC_ was calculated by dividing the molar absorptivity of the tested compound (**21a**–**l**, **25a**–**b**) by that of Trolox.

### 3.3. UV-Vis Spectroscopic Studies

UV-Vis spectra were recorded using the mentioned UV–Vis spectrophotometer at 25 °C. Titration experiments were performed by sequential additions of 0.5–12 μL of metal ion solution (1 mM stock solution of ammonium iron(II) sulfate hexahydrate or copper(II) chloride, freshly made in 0.1 M HCl) to the same 3 mL compound solution in a quartz cuvette (10 μM prepared from 1 mM stock solution in MeOH). The mixture was equilibrated at 25 °C for 10 min. All titrations were performed in 20 mM KPB buffer at pH 7.2 [35].

## 4. Conclusions

Two series of 2-substituted quinazolin-4(3*H*)-ones **21a**–**l** and **25a**–**b** were synthesized from anthranilamide (**20**) and corresponding aldehydes (**19a**–**l**). All synthesized compounds were evaluated for their antioxidant properties in three different methods, namely DPPH, ABTS and TEAC_CUPRAC_ assays. We found that the ABTS and TEAC_CUPRAC_ assays are more appropriate for antioxidant activity evaluation of 2-substituted quinazolin-4(3*H*)-ones. To gain antioxidant activity, the presence of at least one hydroxyl group on the aromatic substituent at position 2 of the main quinazolin-4(3*H*)-ones scaffold is required. The addition of a methoxy substituent or the second hydroxyl group in the ortho or para position relative to the hydroxyl group significantly increases the antioxidant activity. The most potent antioxidants from the first series are 2,3-, 2,5 and 3,4-dihydroxy derivatives **21e**, **21g** and **21h**, respectively. The second series represent two compounds with additional ethylene linker between quinazolinone ring and phenolic substituent, namely 4-hydroxystyryl derivatives **25a** and **25b**, which are more potent antioxidants than 4-hydroxyphenyl counterparts **21d** and **21l**. In addition to high antioxidant activity, quinazolinones **21e** and **21h** also exhibited significant metal-chelating properties.

## Figures and Tables

**Table 1:** Antioxidant activity measurements of synthesized quinazolin-4(3*H*)-ones **21a**–**l** and **25a**–**b**.

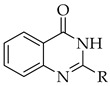		Antioxidant Activity		
		DPPH ^1^	ABTS ^2^	CUPRAC
Compound	Substituent (R)	EC_50_ (µM)	EC_50_ (µM)	TEAC_CUPRAC_
**21a**	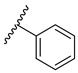	N.A. ^3^	N.A.	N.A.
**21b**	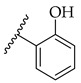	N.A. ^3^	26.7 ± 2.0	0.0345
**21c**	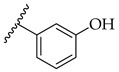	N.A. ^3^	23.0 ± 1.1	0.0191
**21d**	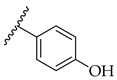	N.A. ^3^	69.9 ± 0.9	0.0315
**21e**	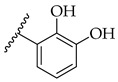	7.5 ± 0.5	9.04 ± 0.44	3.46
**21f**	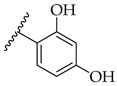	936.3 ± 47.1	8.38 ± 0.12	0.586
**21g**	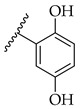	7.4 ± 0.2	8.36 ± 0.38	2.62
**21h**	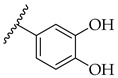	7.2 ± 0.2	10.40 ± 0.26	2.74
**21i**	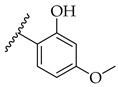	N.D. ^4^	20.1 ± 1.0	0.0906
**21j**	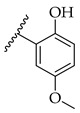	130.0 ± 37.7	17.4 ± 0.7	1.31
**21k**	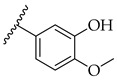	N.D. ^4^	16.7 ± 1.8	0.792
**21l**	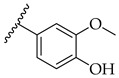	527.7 ± 15.1	15.3 ± 1.3	0.815
**25a**	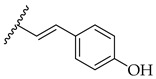	N.D.^4^	22.5 ± 1.1	0.539
**25b**	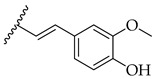	32.4 ± 1.2	6.67 ± 0.62	1.01

^1^ Positive control: α-tocopherol, EC_50_ = 14.2 ± 0.7 µM. ^2^ Positive control: trolox, EC_50_ = 17.6 ± 0.4 µM. ^3^ N.A. = not active (100% of DPPH, 0% of radical scavenging activity). ^4^ N.D. = EC_50_ could not be determined, % of radical scavenging at 500 μM was lower than 30%.

## Data Availability

Data is contained within the article and supplementary material. Other data are available on request from the corresponding author.

## Supplementary Materials

The following are available online. Supporting Information data include Figure S1: Spectrophotometric titration of 10 μM **21a** with Cu^2+^ (a) and Fe^2+^ (b) (0 (blue), 5 (orange), 10 (gray), 20 (ocher) μM) in 20 mM KPB buffer, pH 7.2, 25 °C), Figure S2: Spectrophotometric titration of 10 μM **21b** with Cu^2+^ (a) and Fe^2+^ (b) (0 (blue), 5 (orange), 10 (gray), 20 (ocher) μM) in 20 mM KPB buffer, pH 7.2, 25 °C), Figure S3: Spectrophotometric titration of 10 μM **21c** with Cu^2+^ (a) and Fe^2+^ (b) (0 (blue), 5 (orange), 10 (gray), 20 (ocher) μM) in 20 mM KPB buffer, pH 7.2, 25 °C), Figure S4: Spectrophotometric titration of 10 μM **21d** with Cu^2+^ (a) and Fe^2+^ (b) (0 (blue), 5 (orange), 10 (gray), 20 (ocher) μM) in 20 mM KPB buffer, pH 7.2, 25 °C), Figure S5: Spectrophotometric titration of 10 μM **21e** with Cu^2+^ (a) and Fe^2+^ (b) (0 (blue), 5 (orange), 10 (gray), 20 (ocher) μM) in 20 mM KPB buffer, pH 7.2, 25 °C), Figure S6: Spectrophotometric titration of 10 μM **21f** with Cu^2+^ (a) and Fe^2+^ (b) (0 (blue), 5 (orange), 10 (gray), 20 (ocher) μM) in 20 mM KPB buffer, pH 7.2, 25 °C), Figure S7: Spectrophotometric titration of 10 μM **21g** with Cu^2+^ (a) and Fe^2+^ (b) (0 (blue), 5 (orange), 10 (gray), 20 (ocher) μM) in 20 mM KPB buffer, pH 7.2, 25 °C), Figure S8: Spectrophotometric titration of 10 μM **21h** with Cu^2+^ (a) and Fe^2+^ (b) (0 (blue), 5 (orange), 10 (gray), 20 (ocher) μM) in 20 mM KPB buffer, pH 7.2, 25 °C), Figure S9: Spectrophotometric titration of 10 μM **21i** with Cu^2+^ (a) and Fe^2+^ (b) (0 (blue), 5 (orange), 10 (gray), 20 (ocher) μM) in 20 mM KPB buffer, pH 7.2, 25 °C), Figure S10: Spectrophotometric titration of 10 μM **21j** with Cu^2+^ (a) and Fe^2+^ (b) (0 (blue), 5 (orange), 10 (gray), 20 (ocher) μM) in 20 mM KPB buffer, pH 7.2, 25 °C), Figure S11: Spectrophotometric titration of 10 μM **21k** with Cu^2+^ (a) and Fe^2+^ (b) (0 (blue), 5 (orange), 10 (gray), 20 (ocher) μM) in 20 mM KPB buffer, pH 7.2, 25 °C), Figure S12: Spectrophotometric titration of 10 μM **21l** with Cu^2+^ (a) and Fe^2+^ (b) (0 (blue), 5 (orange), 10 (gray), 20 (ocher) μM) in 20 mM KPB buffer, pH 7.2, 25 °C), Figure S13: Spectrophotometric titration of 10 μM **25a** with Cu^2+^ (a) and Fe^2+^ (b) (0 (blue), 5 (orange), 10 (gray), 20 (ocher) μM) in 20 mM KPB buffer, pH 7.2, 25 °C), Figure S14: Spectrophotometric titration of 10 μM **25b** with Cu^2+^ (a) and Fe^2+^ (b) (0 (blue), 5 (orange), 10 (gray), 20 (ocher) μM) in 20 mM KPB buffer, pH 7.2, 25 °C), Figure S15: Electrospray mass spectrum of solution Cu^2+^ and **21e** (10 μM each, 1:1) in methanol/water (1:1,*v*/*v*), Figure S16: Electrospray mass spectrum of solution Cu^2+^ and **21e** (10 μM each, 1:1) in methanol/water (1:1,*v*/*v*). Peak at *m/z* = 253 belongs to oxidised **21e** orthoquinone.

## Sample Availability

Samples of the compounds are available from the authors.
