# Supplementary material for: Synthesis and Evaluation of Antioxidant Properties of 2-Substituted Quinazolin-4(3H)-ones
Source: Molecules. 2021 Oct 30;26(21):6585. doi: 10.3390/molecules26216585 (PMC8588455; doi:10.3390/molecules26216585)
Supplement: Supplementary file 1 [file molecules-26-06585-s001.zip › molecules-1440906-supplementary.pdf]

Supplementary material on:

Synthesis and evaluation of antioxidant properties of 2-substituted quinazolin-4(*3H*)-ones

Janez Mravljak [1], Lara Slavec [1], Martina Hrast [1] and Matej Sova [1,*]

[1] University of Ljubljana, Faculty of Pharmacy, Aškerčeva 7, SI 1000 Ljubljana, Slovenia; janez.mravljak@ffa.uni-lj.si; martina.hrast@ffa.uni-lj.si
* Correspondence: matej.sova@ffa.uni-lj.si; Tel: +386-1-4769556

TABLE OF CONTENTS

Figures 2
Figure S1 2
Figure S2 3
Figure S3 4
Figure S4 5
Figure S5 6
Figure S6 7
Figure S7 8
Figure S8 9
Figure S9 10
Figure S10 11
Figure S11 12
Figure S12 13
Figure S13 14
Figure S14 15
Figure S15 16
Figure S16 17

Figures

(a)

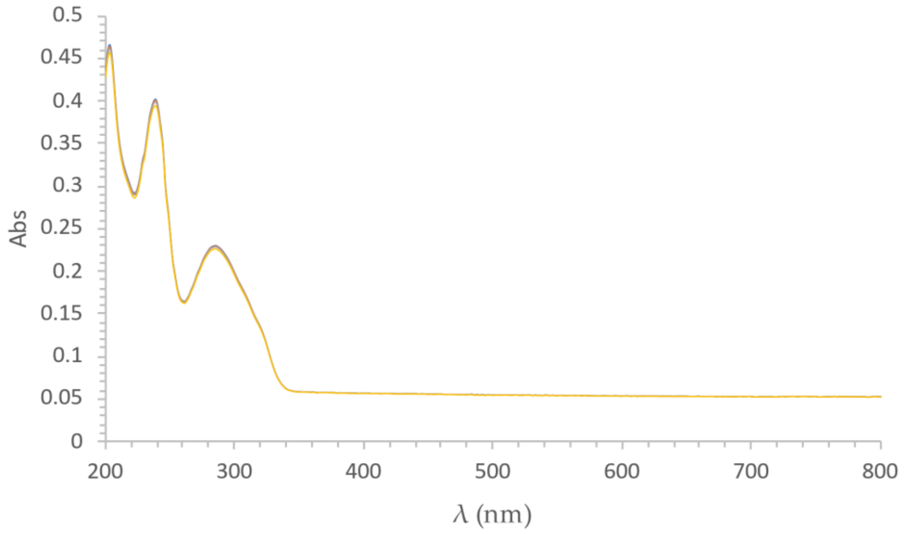

(b)

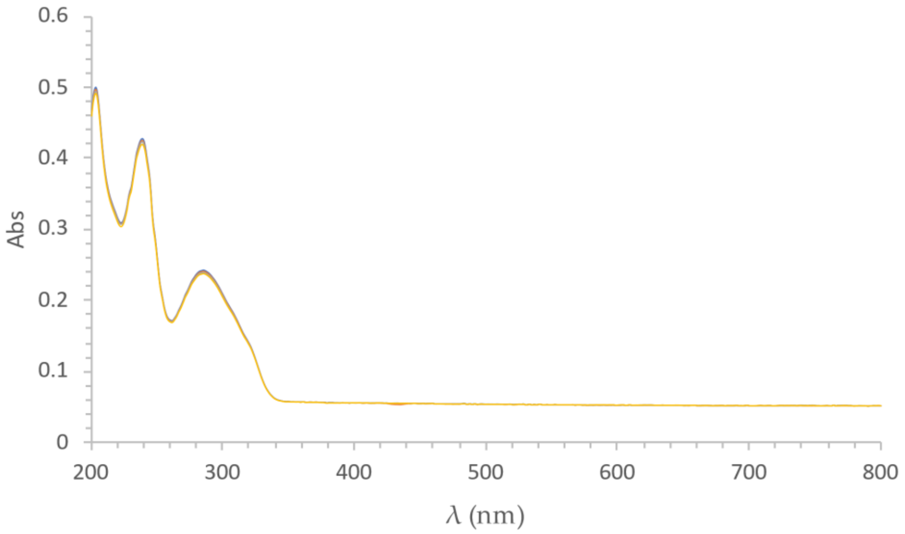

Figure S1. Spectrophotometric titration of 10 μM **21a** with Cu^{2+} (a) and Fe^{2+} (b) (0 (blue), 5 (orange), 10 (gray), 20 (ocher) μM) in 20 mM KPB buffer, pH 7.2, 25 °C).

(a)

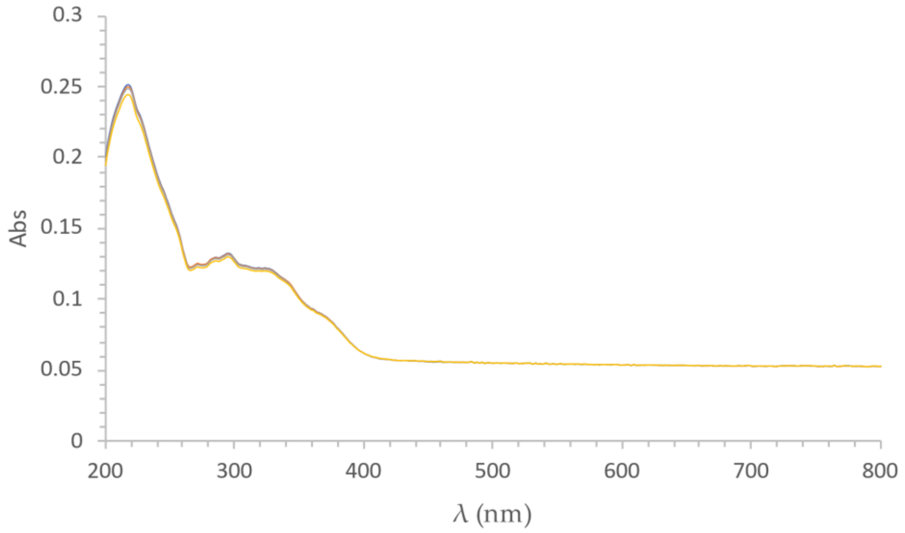

(b)

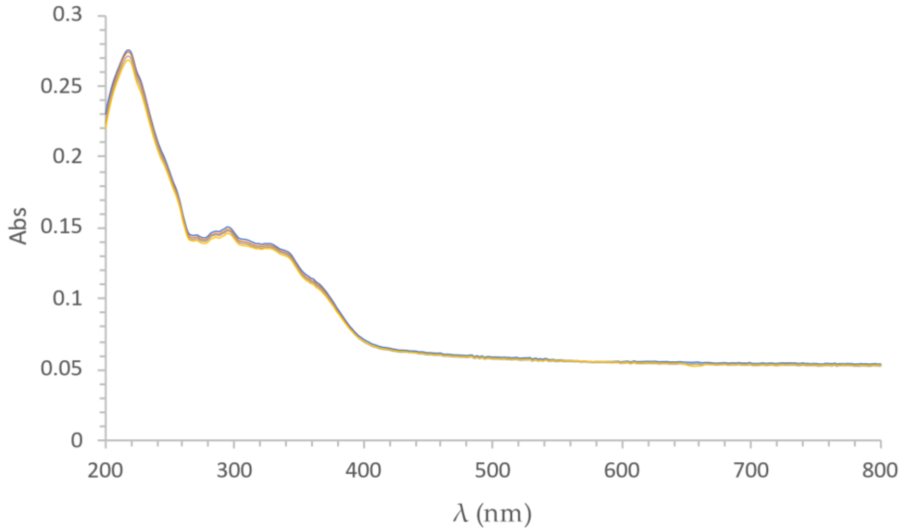

Figure S2. Spectrophotometric titration of 10 μM **21b** with Cu^{2+} (a) and Fe^{2+} (b) (0 (blue), 5 (orange), 10 (gray), 20 (ocher) μM) in 20 mM KPB buffer, pH 7.2, 25 °C).

(a)

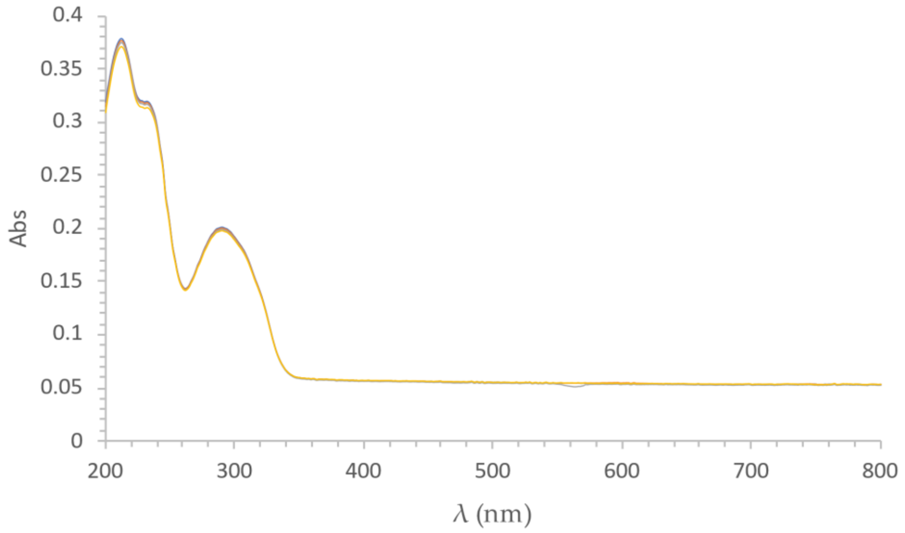

(b)

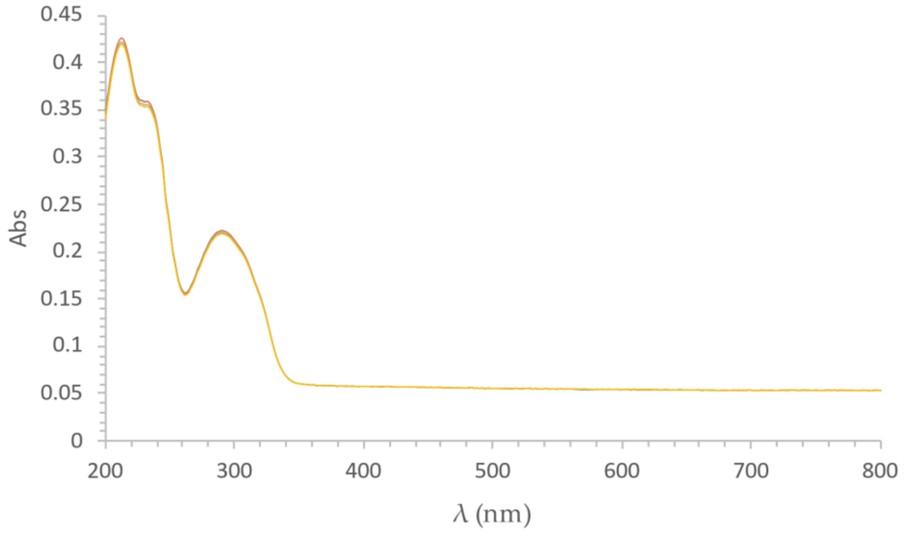

Figure S3. Spectrophotometric titration of 10 μM **21c** with Cu^{2+} (a) and Fe^{2+} (b) (0 (blue), 5 (orange), 10 (gray), 20 (ocher) μM) in 20 mM KPB buffer, pH 7.2, 25 °C).

(a)

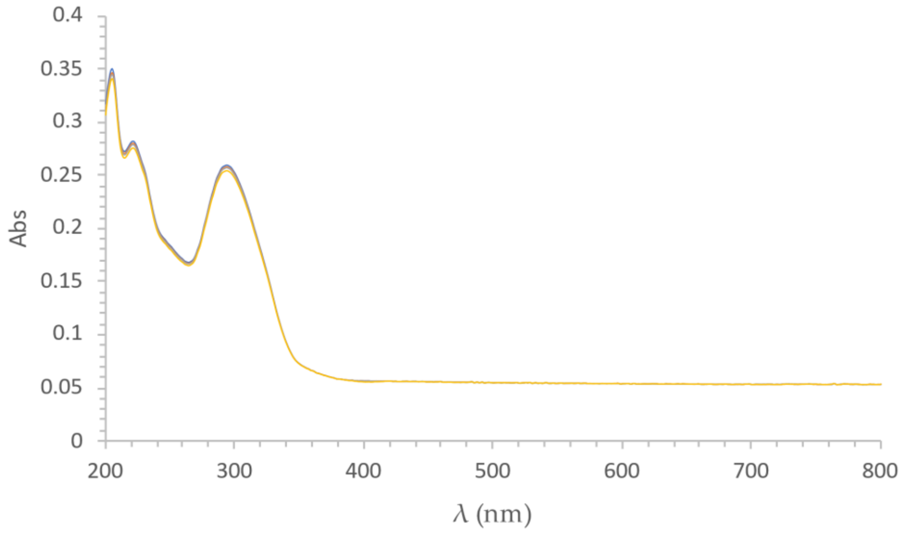

(b)

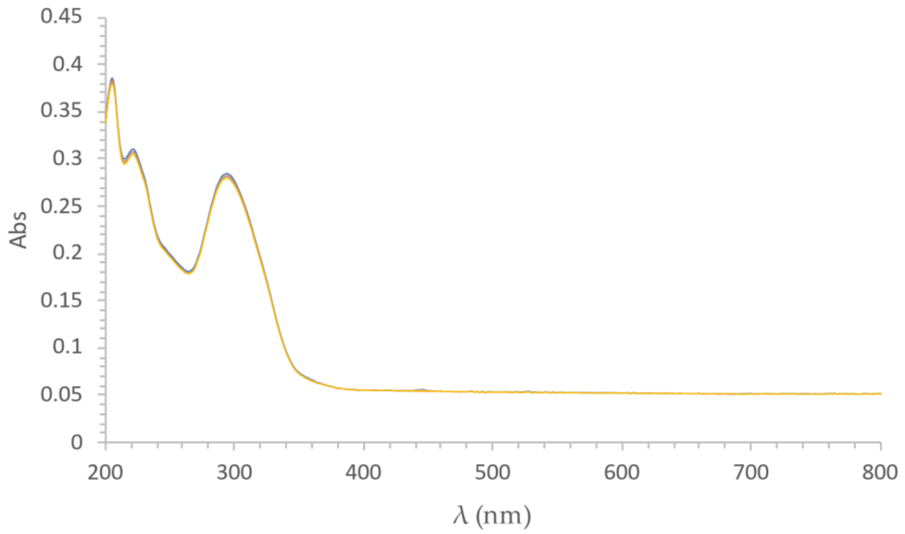

Figure S4. Spectrophotometric titration of 10 μM **21d** with Cu^{2+} (a) and Fe^{2+} (b) (0 (blue), 5 (orange), 10 (gray), 20 (ocher) μM) in 20 mM KPB buffer, pH 7.2, 25 °C).

(a)

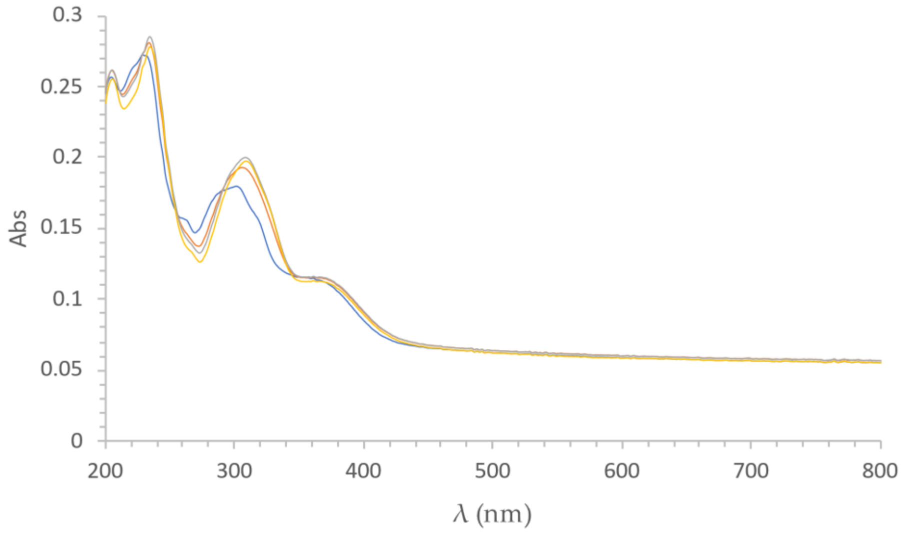

(b)

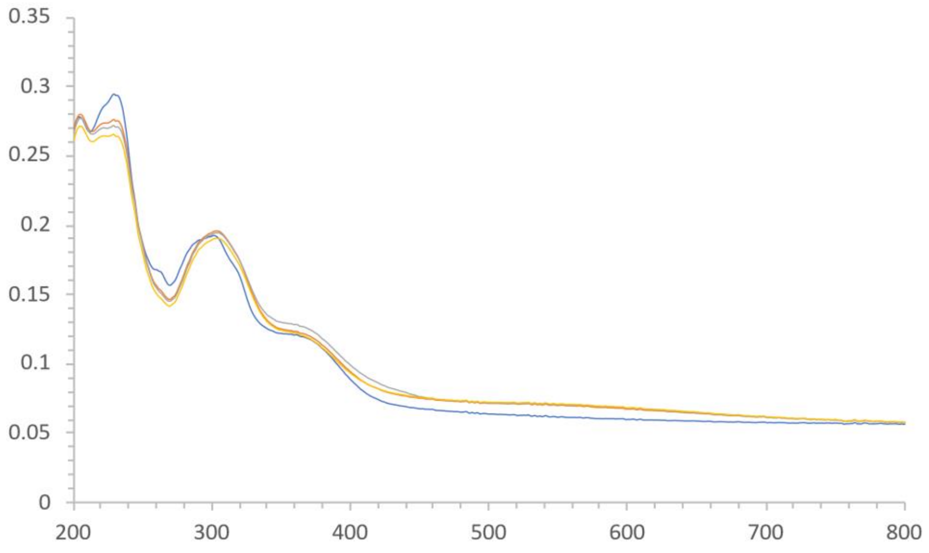

Figure S5. Spectrophotometric titration of 10 μM **21e** with Cu^{2+} (a) and Fe^{2+} (b) (0 (blue), 5 (orange), 10 (gray), 20 (ocher) μM) in 20 mM KPB buffer, pH 7.2, 25 °C).

(a)

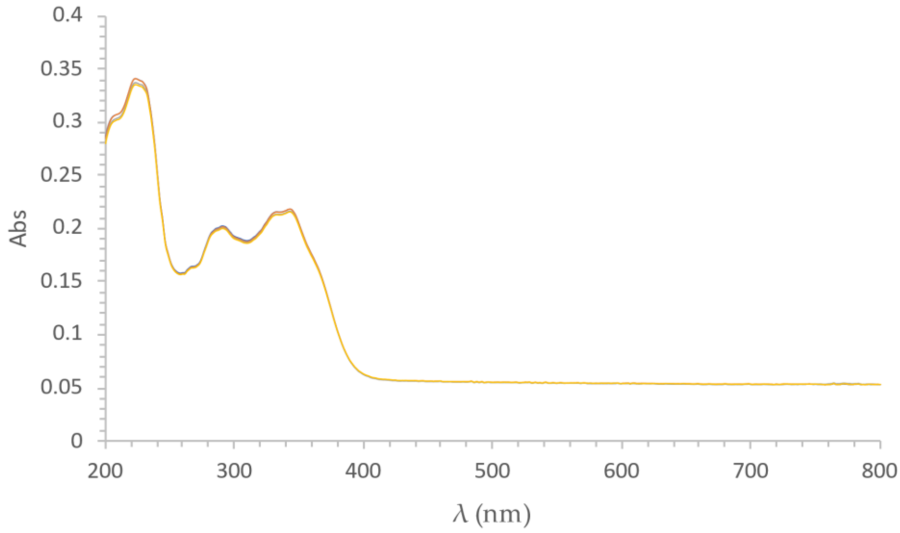

(b)

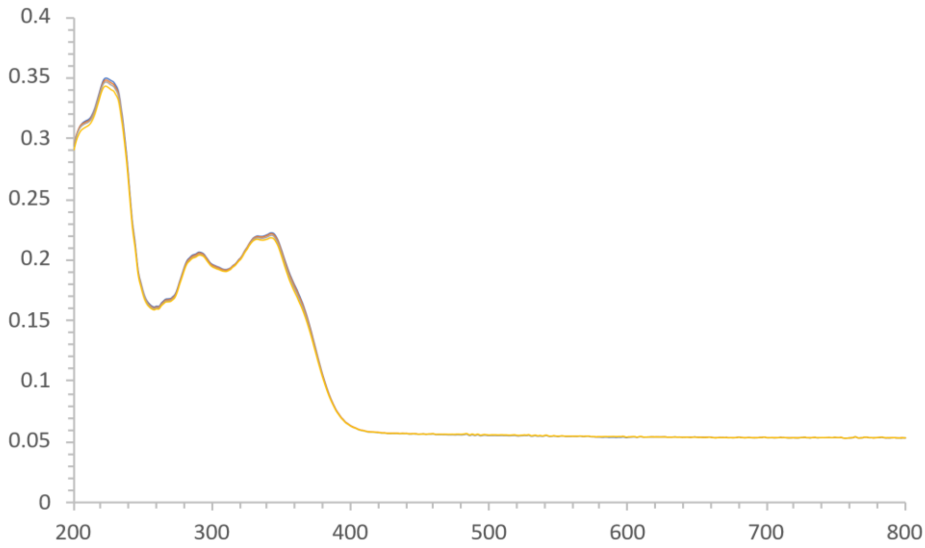

Figure S6. Spectrophotometric titration of 10 μM **21f** with Cu^{2+} (a) and Fe^{2+} (b) (0 (blue), 5 (orange), 10 (gray), 20 (ocher) μM) in 20 mM KPB buffer, pH 7.2, 25 °C).

(a)

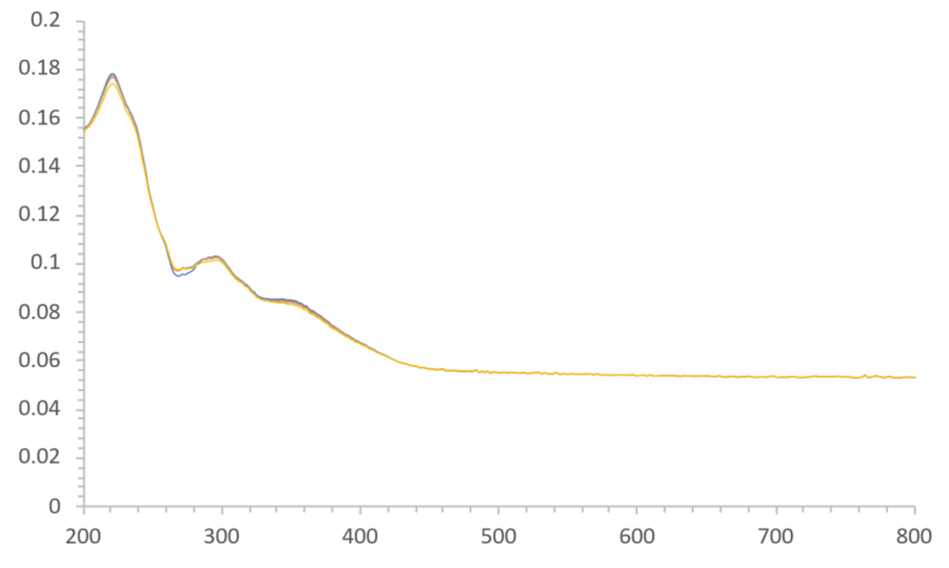

(b)

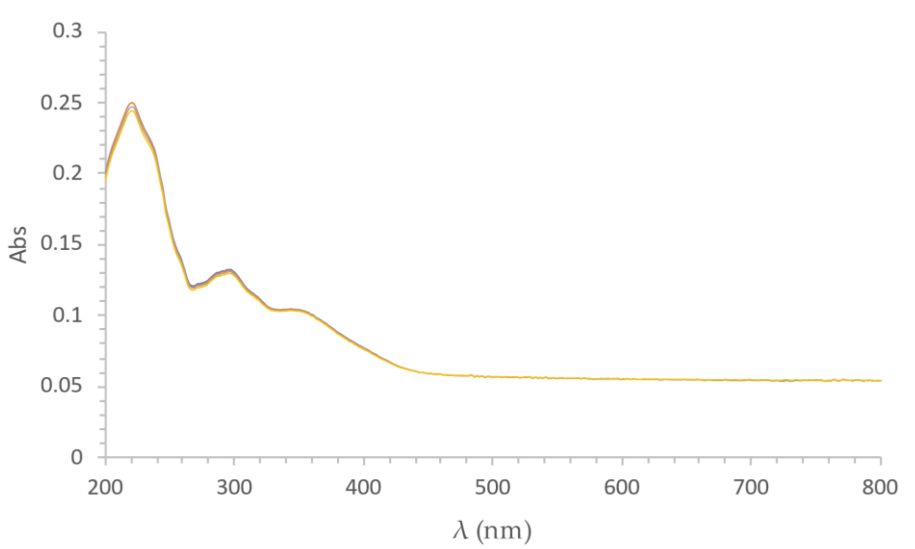

Figure S7. Spectrophotometric titration of 10 μM **21g** with Cu^{2+} (a) and Fe^{2+} (b) (0 (blue), 5 (orange), 10 (gray), 20 (ocher) μM) in 20 mM KPB buffer, pH 7.2, 25 °C).

(a)

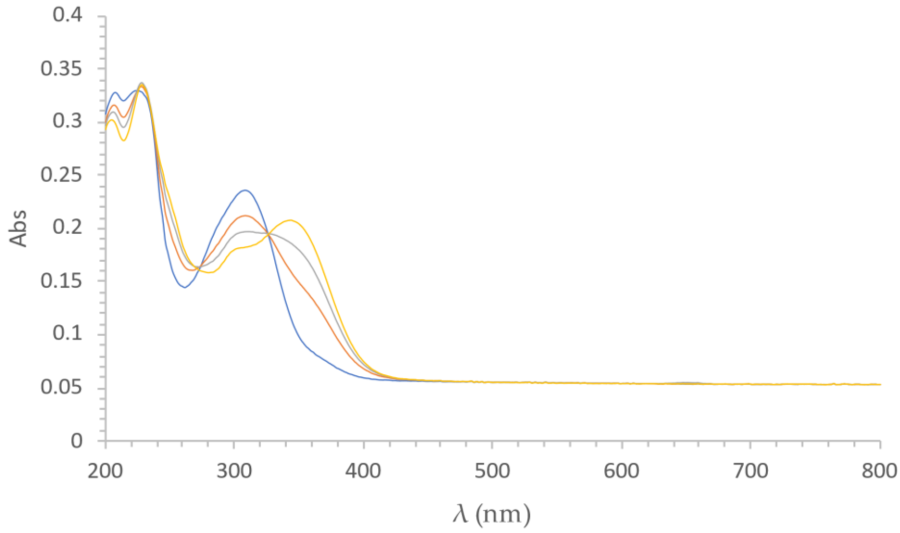

(b)

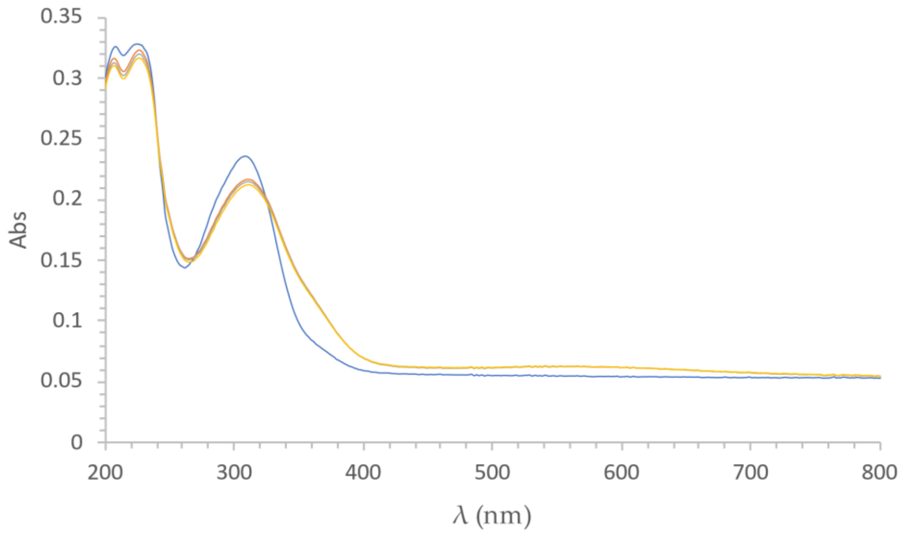

Figure S8. Spectrophotometric titration of 10 μM **21h** with Cu^{2+} (a) and Fe^{2+} (b) (0 (blue), 5 (orange), 10 (gray), 20 (ocher) μM) in 20 mM KPB buffer, pH 7.2, 25 °C).

(a)

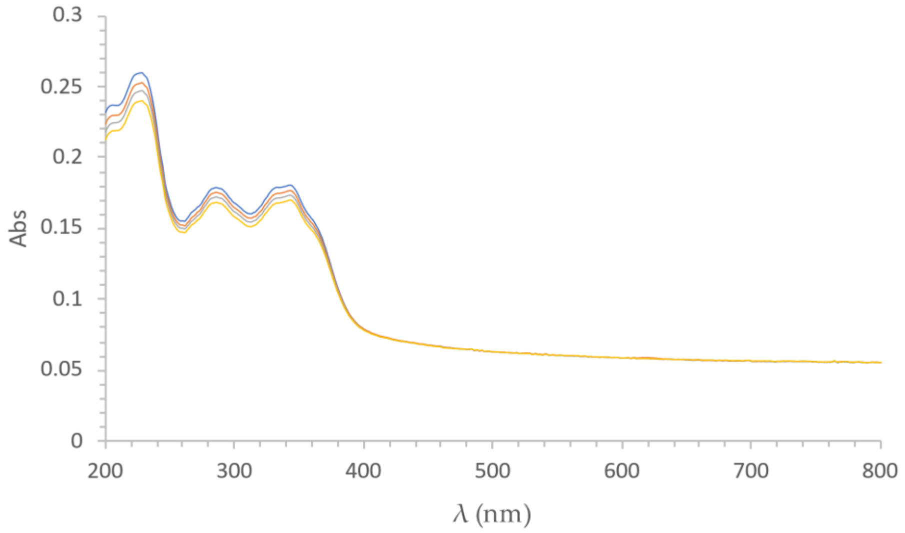

(b)

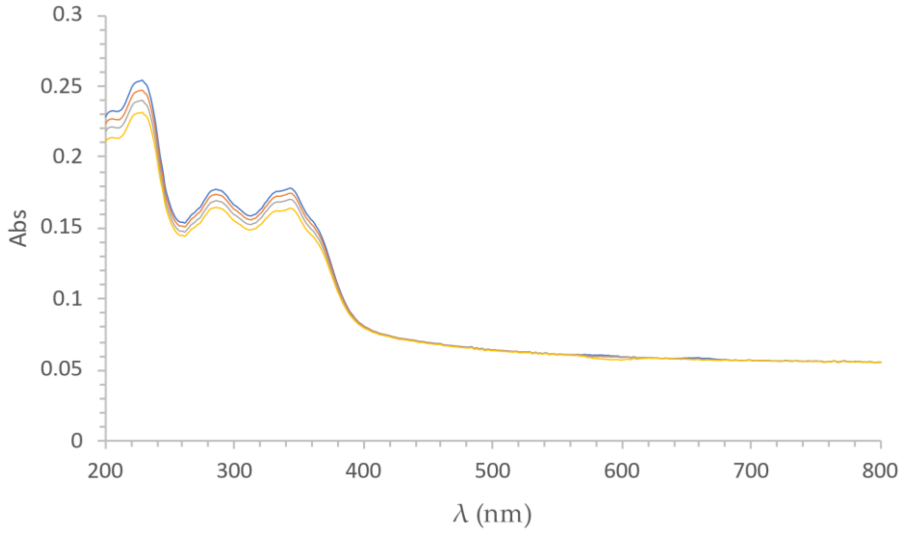

Figure S9. Spectrophotometric titration of 10 μM **21i** with Cu^{2+} (a) and Fe^{2+} (b) (0 (blue), 5 (orange), 10 (gray), 20 (ocher) μM) in 20 mM KPB buffer, pH 7.2, 25 °C).

(a)

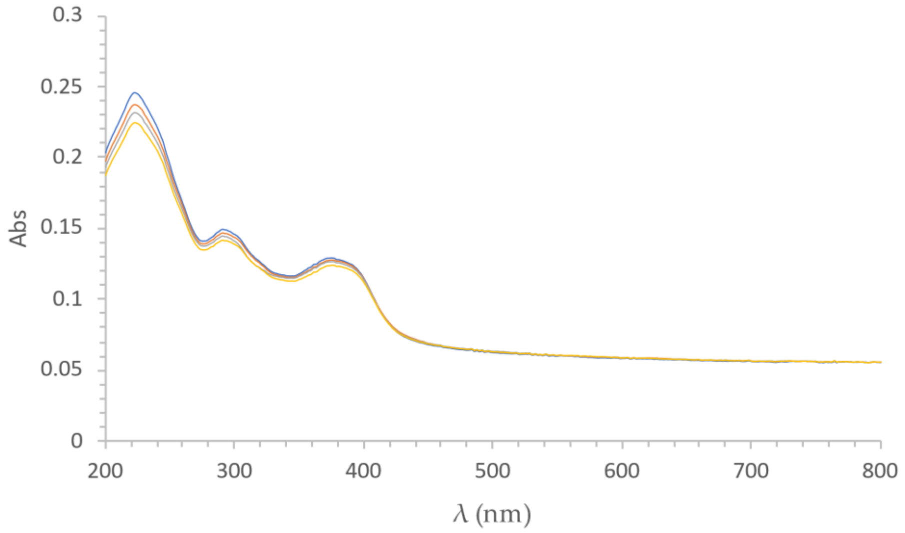

(b)

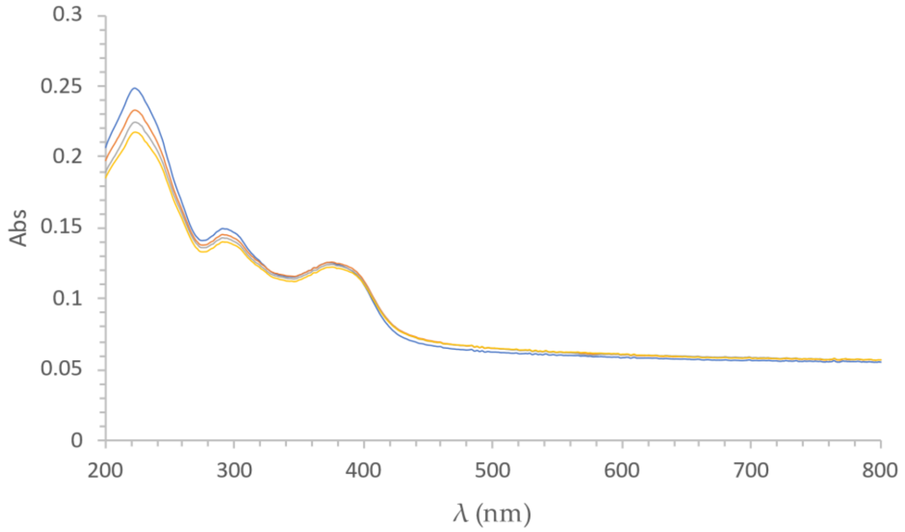

Figure S10. Spectrophotometric titration of 10 μM **21j** with Cu^{2+} (a) and Fe^{2+} (b) (0 (blue), 5 (orange), 10 (gray), 20 (ocher) μM) in 20 mM KPB buffer, pH 7.2, 25 °C).

(a)

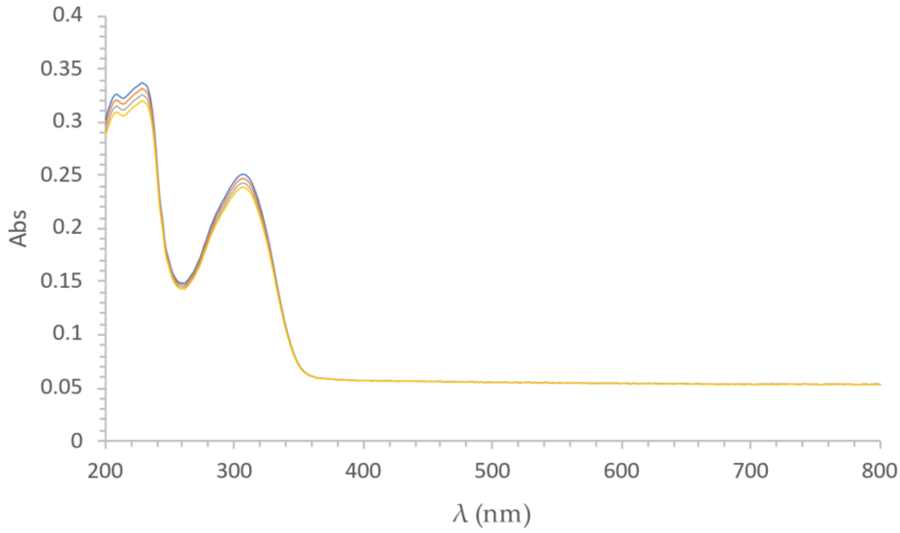

(b)

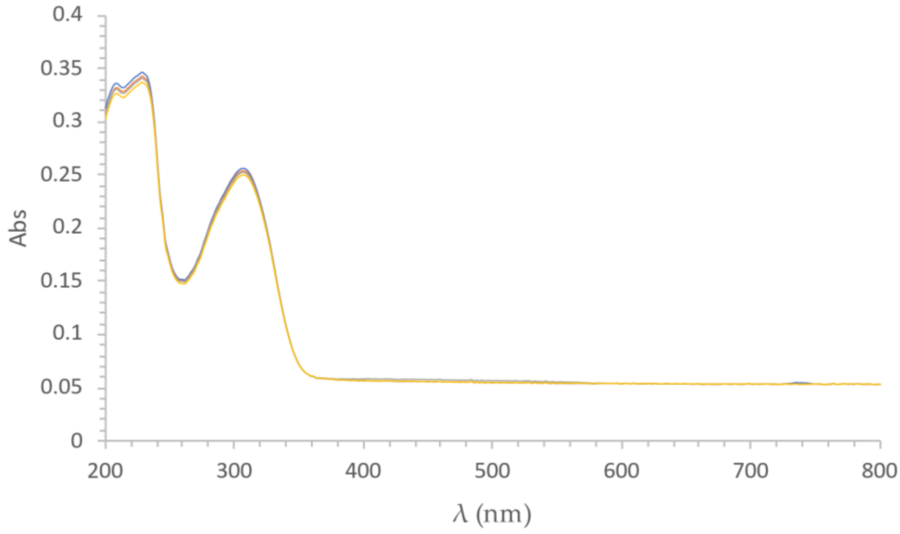

Figure S11. Spectrophotometric titration of 10 μM **21k** with Cu^{2+} (a) and Fe^{2+} (b) (0 (blue), 5 (orange), 10 (gray), 20 (ocher) μM) in 20 mM KPB buffer, pH 7.2, 25 °C).

(a)

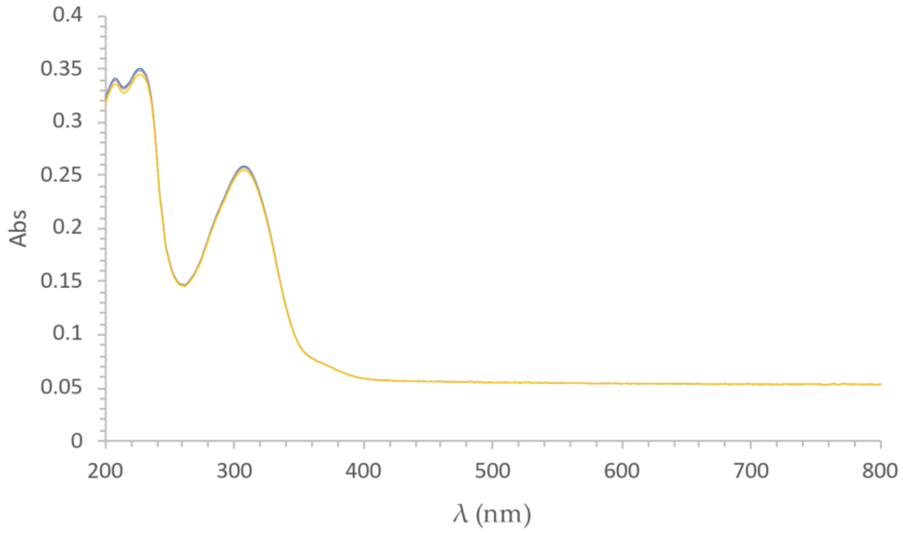

(b)

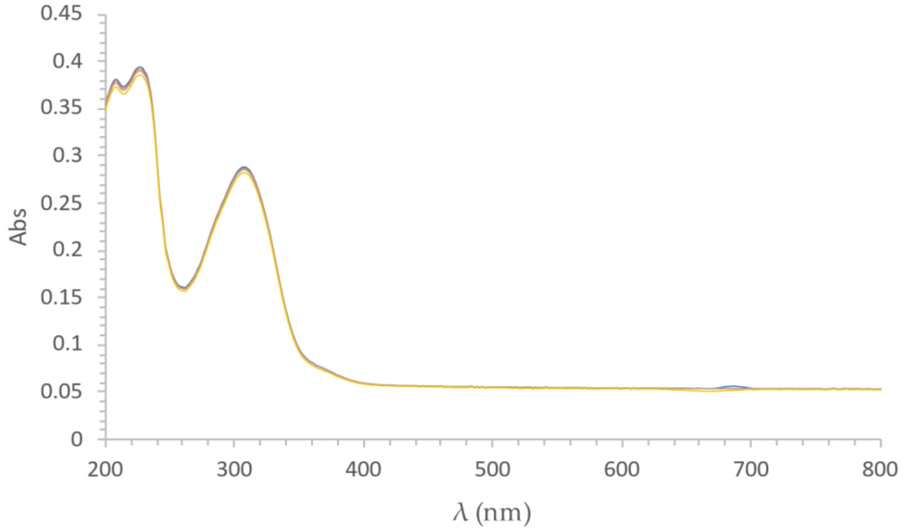

Figure S12. Spectrophotometric titration of 10 μM **21l** with Cu^{2+} (a) and Fe^{2+} (b) (0 (blue), 5 (orange), 10 (gray), 20 (ocher) μM) in 20 mM KPB buffer, pH 7.2, 25 °C).

(a)

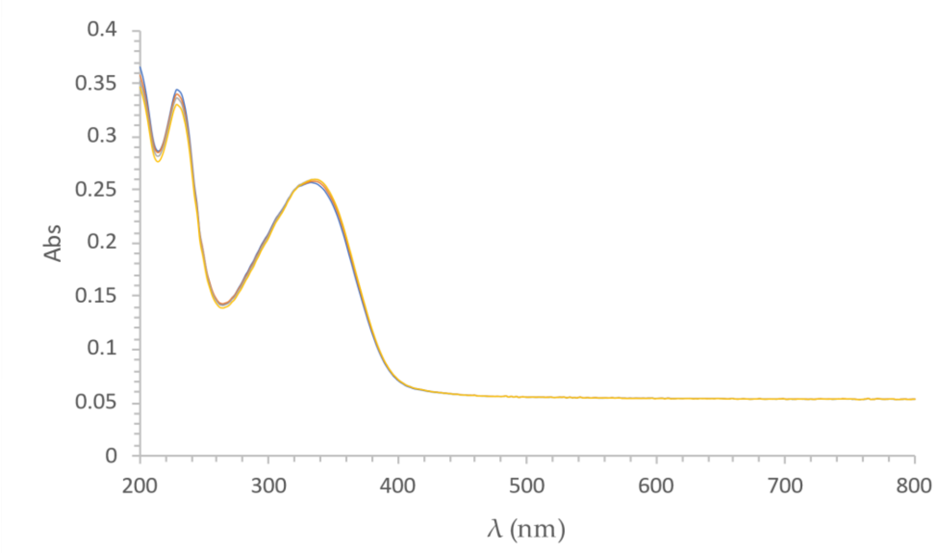

(b)

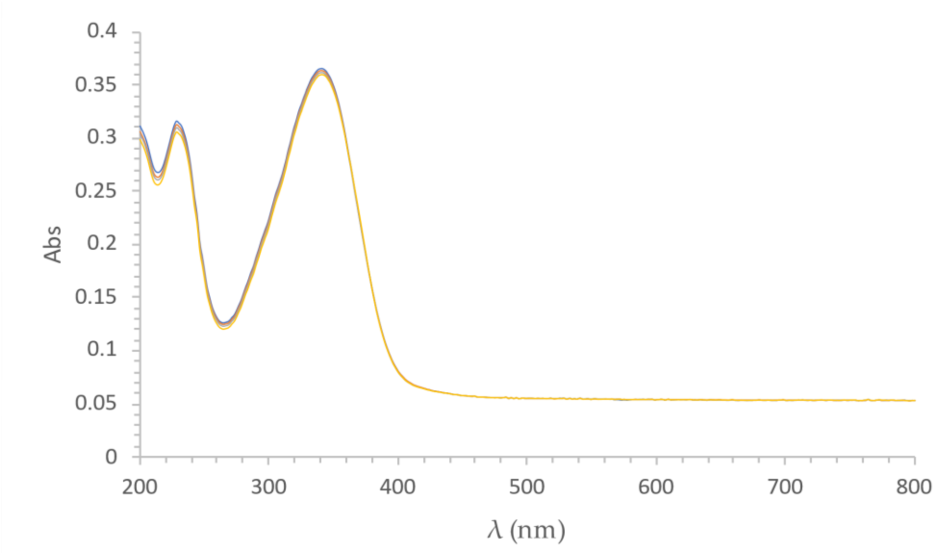

Figure S13. Spectrophotometric titration of 10 μM **25a** with Cu^{2+} (a) and Fe^{2+} (b) (0 (blue), 5 (orange), 10 (gray), 20 (ocher) μM) in 20 mM KPB buffer, pH 7.2, 25 °C).

(a)

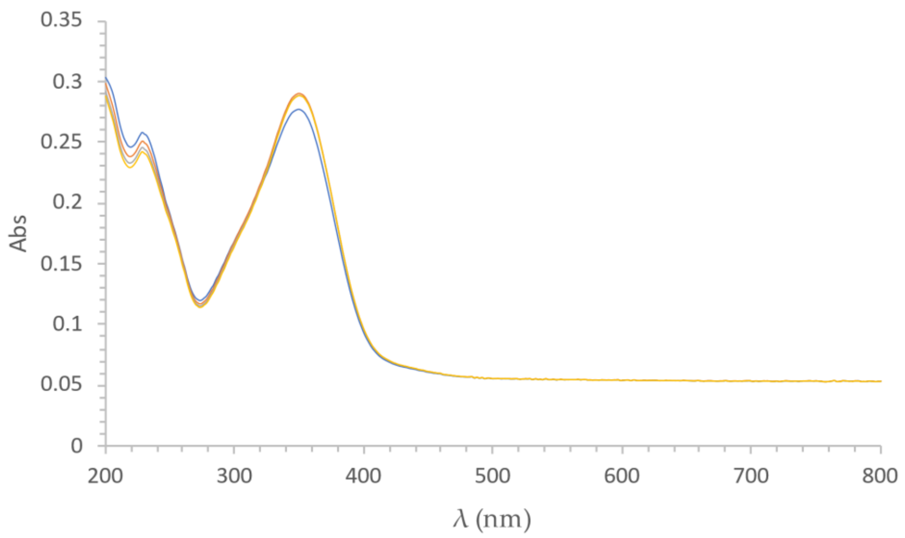

(b)

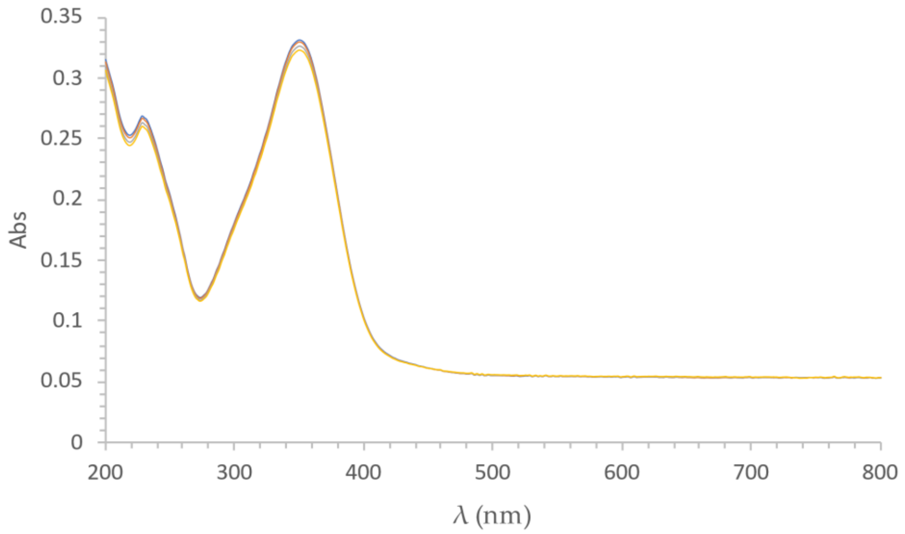

Figure S14. Spectrophotometric titration of 10 μM **25b** with Cu^{2+} (a) and Fe^{2+} (b) (0 (blue), 5 (orange), 10 (gray), 20 (ocher) μM) in 20 mM KPB buffer, pH 7.2, 25 °C).

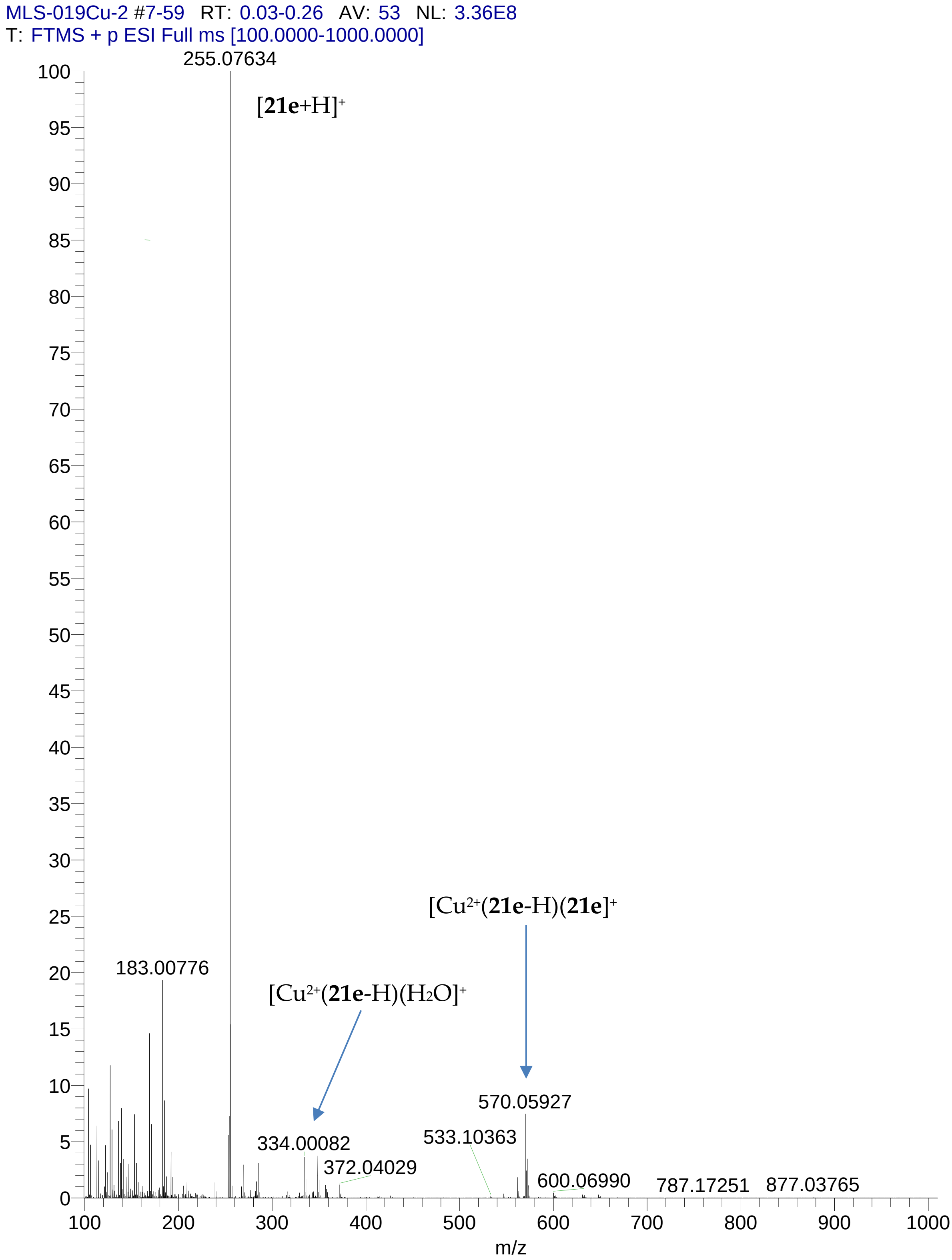

Figure S15. Electrospray mass spectrum of solution Cu^{2+} and **21e** (10 μM each, 1 : 1) in methanol/water (1 : 1, v/v).

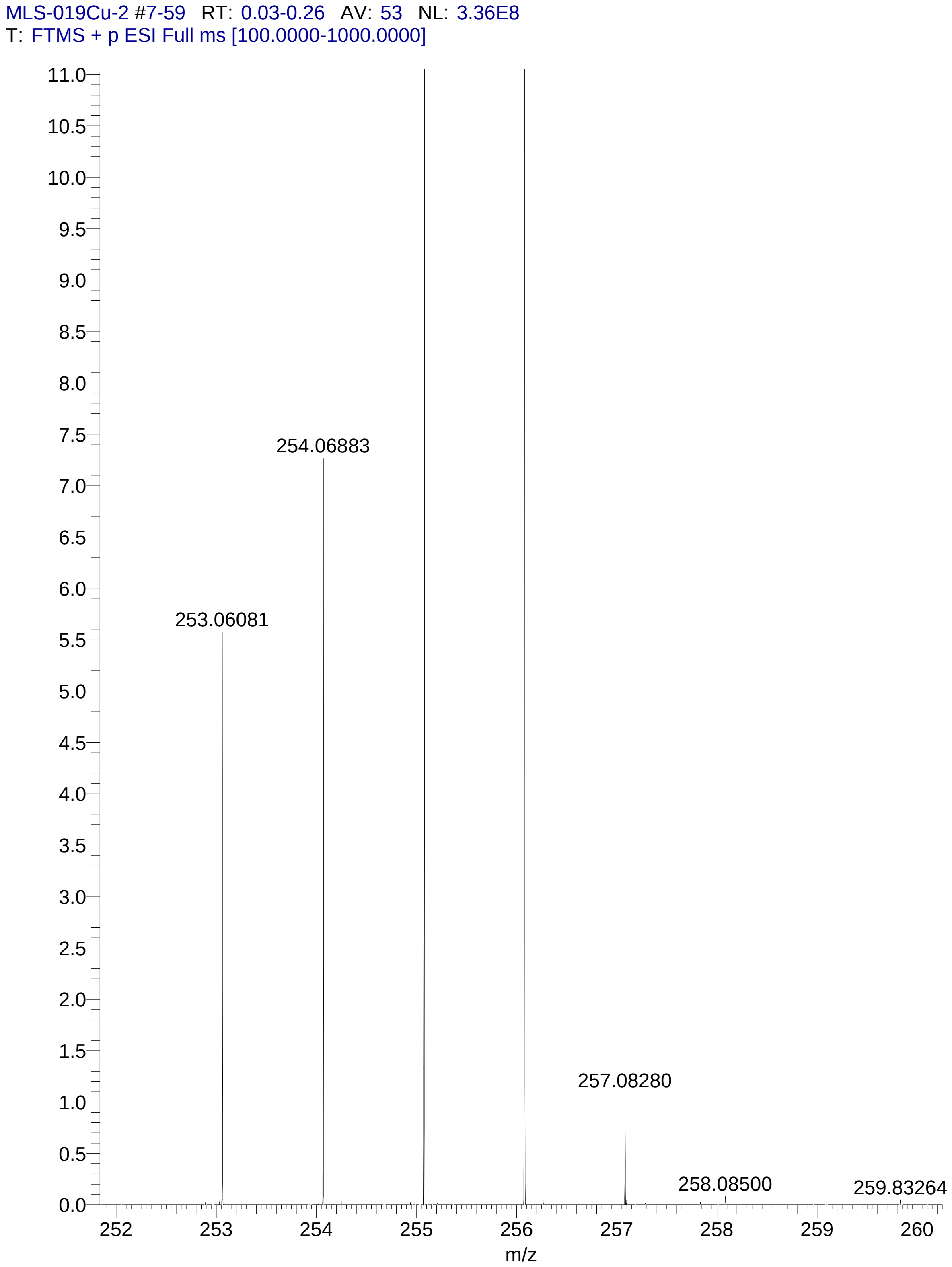

Figure S16. Electrospray mass spectrum of solution Cu^{2+} and **21e** (10 μM each, 1 : 1) in methanol/water (1 : 1, v/v). Peak at *m*/*z* = 253 belongs to oxidised **21e** orthoquinone.
